# A Novel Class of Dual-Acting DCH-CORMs Counteracts Oxidative Stress-Induced Inflammation in Human Primary Tenocytes

**DOI:** 10.3390/antiox10111828

**Published:** 2021-11-18

**Authors:** Federico Appetecchia, Sara Consalvi, Emanuela Berrino, Marialucia Gallorini, Arianna Granese, Cristina Campestre, Simone Carradori, Mariangela Biava, Giovanna Poce

**Affiliations:** 1 Department of Chemistry and Technologies of Drug, Sapienza University of Rome, piazzale A. Moro 5, 00185 Rome, Italy; federico.appetecchia@uniroma1.it (F.A.); Sara.consalvi@uniroma1.it (S.C.); emanuela.berrino@uniroma1.it (E.B.); arianna.granese@uniroma1.it (A.G.); 2 Department of Pharmacy, “G. d’Annunzio” University of Chieti-Pescara, Via dei Vestini 31, 66100 Chieti, Italy; marialucia.gallorini@unich.it (M.G.); cristina.campestre@unich.it (C.C.)

**Keywords:** CO-releasing molecules, tenocytes, PGE_2_, 1,5-diarylpyrrole, 1,5-diarylpyrazole, carbon monoxide

## Abstract

Carbon monoxide (CO) can prevent cell and tissue damage by restoring redox homeostasis and counteracting inflammation. CO-releasing molecules (CORMs) can release a controlled amount of CO to cells and are emerging as a safer therapeutic alternative to delivery of CO in vivo. Sustained oxidative stress and inflammation can cause chronic pain and disability in tendon-related diseases, whose therapeutic management is still a challenge. In this light, we developed three small subsets of 1,5-diarylpyrrole and pyrazole dicobalt(0)hexacarbonyl (DCH)-CORMs to assess their potential use in musculoskeletal diseases. A myoglobin-based spectrophotometric assay showed that these CORMs act as slow and efficient CO-releasers. Five selected compounds were then tested on human primary-derived tenocytes before and after hydrogen peroxide stimulation to assess their efficacy in restoring cell redox homeostasis and counteracting inflammation in terms of PGE_2_ secretion. The obtained results showed an improvement in tendon homeostasis and a cytoprotective effect, reflecting their activity as CO-releasers, and a reduction of PGE_2_ secretion. As these compounds contain structural fragments of COX-2 selective inhibitors, we hypothesized that such a composite mechanism of action results from the combination of CO-release and COX-2 inhibition and that these compounds might have a potential role as dual-acting therapeutic agents in tendon-derived diseases.

## 1. Introduction

CO-releasing molecules (CORMs) can release carbon monoxide (CO) either spontaneously, enzymatically, or triggered by an external stimulus [1]. Their therapeutic potential relies on the release of a limited amount of CO. Along with NO and H_2_S, CO is the third small signaling molecule and is produced endogenously by enzymes of the Heme Oxygenase (HO) class through heme oxidative degradation. The expression of the HO inducible isoform (HO-1) is triggered by cell responses toward oxidative stress and inflammation and results in cyto- and tissue protection. HO-1 metabolites, including CO, are important in restoring redox homeostasis and resolution of inflammation, and it has been widely demonstrated that the HO-1/CO axis can help to prevent cellular and tissue damage. Therefore, the manipulation of the HO-1/CO system is an attractive strategy to treat conditions linked to oxidative-stress-induced inflammation, such as lung hyper-inflammation in cystic fibrosis, sepsis and modulation of chronic pain [2,3,4,5,6,7]. The chemistry of CO is unique: unlike NO and H_2_S that react indiscriminately with intracellular targets, CO offers the advantage of binding only to transition metals in a low oxidation state. Such preferential reactivity, along with its greater stability, makes it a more versatile candidate for the development of gaseous-based pharmaceuticals [8]. Indeed, gaseous CO has great potential as a therapeutic tool and has been found beneficial in the treatment of several inflammatory, cardiovascular, and neurological diseases [9,10,11,12]. For low-dose CO inhalation, the feasibility of the first clinical trials has been recently assessed [13]. However, the accurate delivery of gaseous CO to its molecular targets through inhalation is challenging, and inhalation therapy is hampered by CO low bioavailability and high affinity to hemoglobin, with consequent toxicity [14]. In this scenario, CORMs have emerged as a safer and attractive therapeutic strategy to deliver a controlled amount of CO to cells. To date, most of the developed CORMs are metal carbonyl complexes (MCCs) [15,16]. Indeed, considering the preferential reactivity of CO for transition metals in a low oxidation state, organometallic complexes have emerged as suitable models to safely deliver CO in vivo and generate innovative therapeutic agents with reasonable pharmacological properties. These molecules have an octahedral shape with six ligands around a central metal and can release CO spontaneously, mainly through hydrolysis in biological buffers. Romão and co-workers [17] introduced a conceptual model to rationalize and improve the design of MCCs with appropriate pharmaceutical properties. This model comprises three portions: (i) a metal core, which accounts for toxicity and the main properties of the MCC; (ii) a coordination-sphere, which influences the electronic density around the metal, tuning the stability and the chemical behavior of the whole complex and triggering CO release under specific conditions; and (iii) a drug-sphere, obtained through modulation of the distal sites of the metal complexes and accounting for pharmacological properties and drug-likeness.

The choice of the transition metal is crucial to design metal-based CORMs. CORMs containing an atom of cobalt (dicobalt(0)hexacarbonyl complexes, DCH) are innovative CO-releasing agents with interesting biological features and good CO-release kinetics [18,19,20,21,22,23,24,25]. The DCH metal core is a hexacarbonyl dicobalt moiety (Co_2_CO_6_) coordinated through an alkyne bond, which is in turn linked to the drug sphere. One of the main advantages of DCH-CORMs is their synthetic accessibility. Indeed, this highly versatile chemical scaffold is easy to synthesize, facilitating the chemical manipulation of the drug sphere. A series of dual acting DCH-CORMs-carbonic anhydrase inhibitors (CAI-CORMs) have very recently shown promising anti-inflammatory properties under oxidative-stress conditions in different oxidative-based disease models [22,23]. Interestingly, Gallorini et al. [26] demonstrated that some of these compounds were able to differentially modulate inflammation and counteract the H_2_O_2_-induced stress in rotator-cuff-derived human tenocytes, which activate the nuclear factor erythroid 2 [NF-E2]-related factor 2 (Nrf2)/HO-1/CO pathway to mitigate oxidative stress. It has also been reported that sustained oxidative stress causes aberrant cytokine secretion in a model of rotator cuff disease (RCD) in vitro [27]. Oxidative stress endurance and, consequently, inflammation occurrence, are considered the major factors causing the failure of tendon healing in clinical practice and can lead to chronic pain and disability. Moreover, the benefits of non-steroidal anti-inflammatory drug (NSAID)-based therapy in the acute phase are broadly accepted, but their use in chronic tendon-related diseases is still controversial [28]. Therefore, an innovative therapeutic approach for the treatment of tendon-derived diseases is urgently needed, as their therapeutic management remains a challenge.

**Figure 1:**
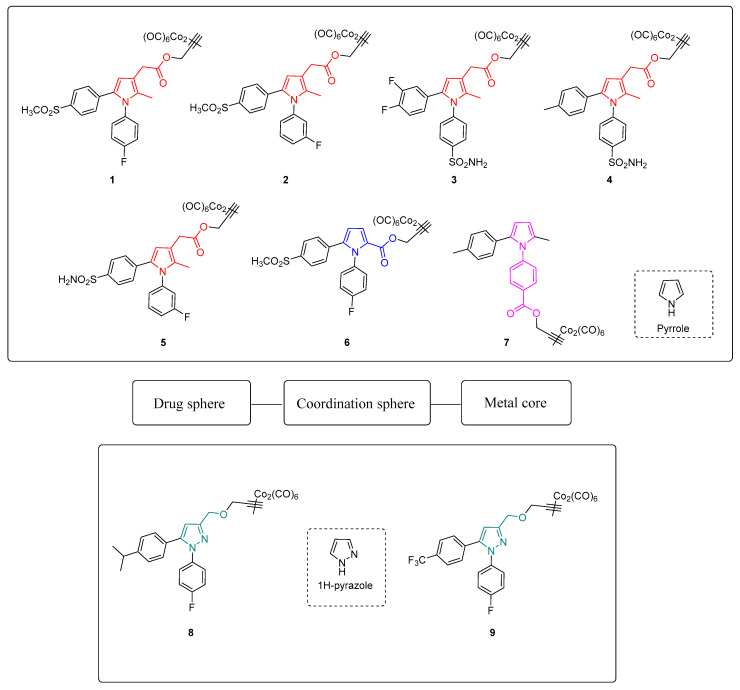
Chemical structures and conceptual model of compounds **1**–**9**.

In this light, we synthesized a small set of 1,5-diarylpyrrole and 1,5-diarylpyrazole-based DCH-CORMs linked through a propargylic chain (compounds **1**–**9**, Figure 1). According to the Romão model, the first aim of this study was to analyze the influence of different electronic and steric properties of the drug sphere on the CO release rate. Five selected compounds (**1**–**5**) were then tested on human primary tendon-derived cells stimulated with a low concentration of hydrogen peroxide (H_2_O_2_), using the NSAID Meloxicam as a reference compound. The present work aims to assess their efficacy in restoring cell redox homeostasis and counteracting inflammation in terms of PGE_2_ secretion and at investigating their potential use in vitro to manage musculoskeletal diseases.

## 2. Materials and Methods

### 2.1. Chemistry

All chemicals used were obtained from commercial sources (Merck, Acros, Syngene) and were used as supplied without further purification. Merck silica gel 60 (230–400 mesh) and Merck aluminum oxide (activity II–III, according to Brockmann) were used for chromatographic columns with the indicated solvents. Merck TLC plates (silica gel 60 F254 and aluminum oxide F254) were used to monitor all operations, and then compounds were visualized under UV light (254 and 365 nm) and/or stained with the relevant reagents. The yields were not optimized and refer to the purified products. ^13^C NMR and ^1^H NMR spectra were recorded on a Bruker Avance III NMR 400 spectrometer with reference to tetramethylsilane (TMS) in the indicated solvent. Chemical shift values are expressed in parts per million (ppm). Coupling constants (*J*) are reported in hertz with signal multiplicities indicated as singlet (s), doublet (d), triplet (t), quadruplet (q) and multiplet (m). When specified, ChemDraw Professional 16.0 was used to generate systematic compound names following IUPAC conventions. Detailed synthetic procedures and spectroscopic data are reported in the Supplementary Materials.

### 2.2. CO-Release Assay

All reagents were of analytical grade and purchased from Merck. Gaseous CO was obtained from Rivoira (Milan, Italy). A Shimadzu UV1900 UV-Vis Spectrophotometer from 275 to 700 nm at the scanning rate of 200 nm/min was used to record UV-Vis absorption spectra in a disposable plastic cuvette (path length 0.44 cm). An Origin Lab software generated second derivative spectra, and the Savitzky–Golay method was applied using 25 data points for the differentiation process. Neither an increase nor a decrease in the number of points caused changes in the wavelength or in the bandwidth. Lyophilized horse heart Mb was dissolved in phosphate buffered saline flushed with N_2_ (PBS, 0.01 M, pH 7.4 to a 20–22 μM final concentration). Two milliliters of this freshly prepared stock solution were placed in a cuvette to record the UV-Vis absorption spectrum of met-Mb. Next, the solutions were divided into two: 10 μL of sodium dithionite (30 mg/mL) were added to the first half (reference) and the UV-Vis spectrum of deoxy-Mb was registered. After that, the solution was flushed with CO gas, and the Mb-CO spectrum was acquired. Sodium dithionite was added to the second half (sample), and a spectrum was recorded. Afterwards, a CORM DMSO solution was added to a final CORM concentration of 3.33 μM and gently mixed. The solution was covered with 300 μL of light mineral oil to avoid CO escaping and oxygenation of Mb, and the absorption spectrum was recorded at t = 0. Spectra were acquired every 30 min for 210 min, keeping the sample at 37 °C. When necessary, a freshly prepared sodium dithionite solution was added. After 210 min, the total Mb concentration at the end of the assay was determined by flushing the sample with CO gas. Mb-CO concentration at each time point was determined as previously reported [23]. Each experiment was replicated three times, and the data were expressed as mean ± SEM.

### 2.3. Cell Culture

Human tenocytes (#TEN-F; ZenBio Inc.; Durham, NC, USA) were maintained in complete alpha-MEM (EuroClone, Milan, Italy) supplemented with 10% of heat-inactivated FBS (Gibco, ThermoFisher Scientific, Waltham, MA, USA) and 1% penicillin/streptomycin (EuroClone, Milan, Italy) at 37 °C and 5% CO_2_ and used from passage 3 up to passage 6.

### 2.4. Cell Treatment

Cells were seeded in 96-well plates (0.5 × 10^4^/well) (ThermoFisher Scientific, Waltham, MA, USA) and left to adhere overnight at 37 °C and 5% CO_2_. In a first set of experiments, tendon-derived cells were treated with increasing concentrations of compounds **1**–**5** in the range 0–100 µM for 24 and 72 h. Compounds were dissolved in DMSO to obtain a 200 mM stock solution, and they were afterwards diluted in complete alpha-MEM (DMSO final concentration = 0.1%) for further analyses. In a second set of experiments, tenocytes were pre-incubated with 100 µM H_2_O_2_ for 3 h. After that, the pre-incubation medium was discarded and replaced with a fresh one containing the proper CORM at increasing concentrations for 24 and 72 h. At the established time points, samples were processed for further analyses.

### 2.5. Cell Metabolic Activity

At the established time points (24 and 72 h), the incubation medium was harvested for further analyses, and complete alpha-MEM containing 0.5 mg/mL MTT (3-[4,5-dimethylthiazol-2-yl]-2,5-diphenyl tetrazolium bromide) (Sigma-Aldrich, St. Louis, MO, USA) was added to each well. Afterwards, cells were incubated for 5 h at 37 °C and cell metabolic activity was measured as already reported [29].

### 2.6. PGE_2_ Secretion

Cell supernatants were collected from the 96-well plates used for the metabolic activity assay (MTT) after 24 and 72 h, and PGE_2_ secretion was analyzed. A commercial ELISA kit (Enzo Life Sciences, Farmingdale, NY, USA) was used to measure the amount (pg/mL) of PGE_2_ in the culture media, according to the manufacturer’s instructions. The PGE_2_ concentration in each sample was determined following a previously reported procedure [30].

## 3. Results and Discussion

### 3.1. Chemistry

Compounds **1**-**9** were easily synthesized in good yields by reacting the terminal alkyne of propargylic intermediates and dicobalt(0)carbonyl octacarbonyl in tetrahydrofuran (THF). Detailed synthetic procedures are reported in the Supporting Information.

### 3.2. CO Release Assay

**Figure 2:**
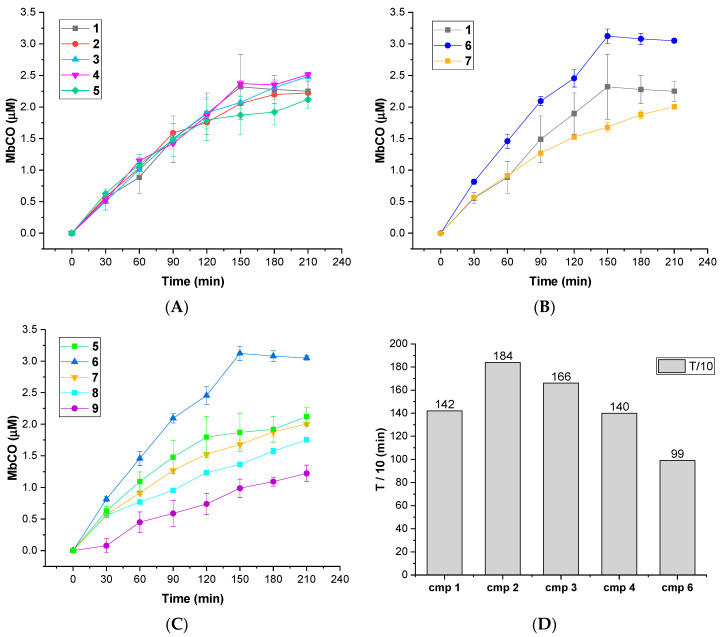
(**A**–**C**): CO-release profiles of compounds **1**–**9** as Mb-CO formed over time (1:6 CORM:Mb ratio); (**D**): *T*_1/10_ values for compounds **1**–**4** and **6** (defined as the time necessary for a CORM solution to produce a Mb-CO concentration of 1/10 of the initial).

**Figure 3:**
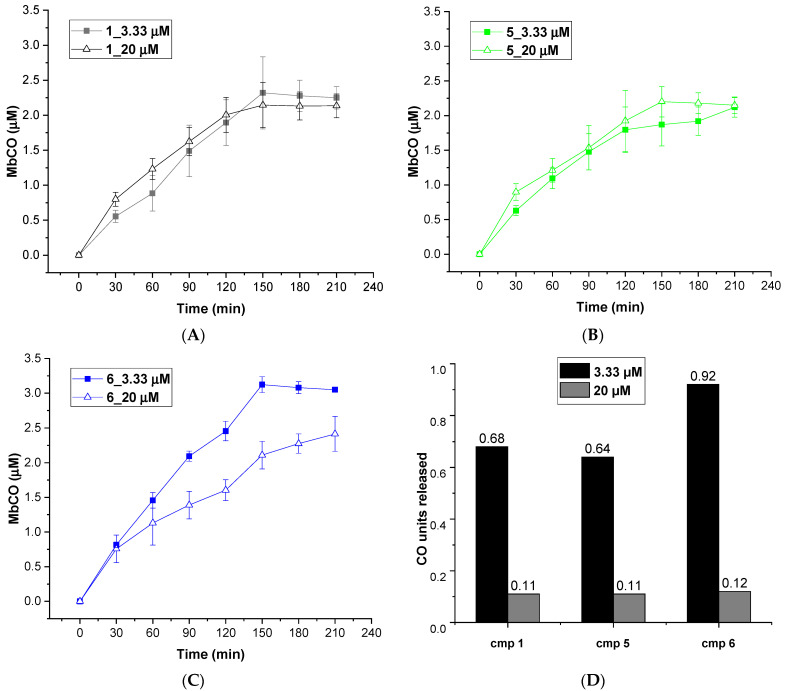
CO release profiles of compounds **1** (**A**), **5** (**B**) and **6** (**C**) analyzed at 1:6 (filled square) and 1:1 (empty triangles) CORM-Mb ratios; (**D**): CO units released by compounds **1**, **5** and **6** after 210 min of incubation working at 1:6 (blue columns) and 1:1 (orange columns) CORM-Mb ratios.

The CO releasing behaviors of compounds **1**–**9** were evaluated through a myoglobin (Mb)-based spectrophotometric assay, considered the gold standard to analyze the CO releasing kinetics and a key criterion to select CORM structures [17,31]. This method analyzes the release of CO from CORMs by following the conversion of deoxy-myoglobin (deoxy-Mb(II)) into CO-myoglobin (CO-Mb(II)) over time by UV-Vis spectroscopy. A 3.3 µM solution of compounds **1**–**9** was incubated with a 20 µM solution of deoxy-Mb (CORM/Mb 1:6 ratio), and a reducing agent (sodium dithionite) was added to prevent oxidation of deoxy-Mb(II) to Met-Mb(III). Changes in the absorption band in the Soret region of deoxy-Mb and Mb-CO were recorded every 30 min for 210 min, and a second derivative approach was applied to clearly discriminate between the three forms of Mb (deoxy-Mb, Mb-CO and Met-Mb). The relative amount of CO produced over time was calculated following a previously reported equation [23,32], and a correction factor was applied to account for Mb degradation induced by sodium dithionite. Concentration values of Mb-CO formed over time by compounds **1**–**9** are reported in Table 1. Their CO-release profiles are shown in Figure 2A–C, along with their *T*_1/10_ values, defined as the time necessary for a CO-RM to produce a concentration of CO-Mb of 1/10 of the initial (Figure 2D). The number of CO units released by CORMs **1**-**9** after 210 min is reported in Table 2. For selected compounds, the assay was performed using a 1:1 CORM/Mb ratio (Figure 3A–D). The aim was to explore their ability to release CO in a less favored condition, considering that CO release from CORMs is stimulated by an excess of Mb.

All the tested compounds were effective CO releasers, with CO release kinetics comparable to previously reported DCH-CORMs (slow CO release up to 210 min) [22,23,33]. The obtained data showed considerable differences among the analyzed compounds, depending on the heterocyclic nucleus and the different alkyne system linked to the DCH group (Table 1).

As shown in Figure 1, compounds **1**–**5** bear a propynyl-pyrrol-3-yl-acetate moiety, compound **6** a propynyl-pyrrole-2-carboxylate motif and compound **7** a propynyl benzoate group at position 1 of the pyrrole ring. Compounds **8** and **9** are characterized by a 3-((prop-2-yn-1-yloxy)methyl)-1*H*-pyrazole scaffold. Derivatives **1**–**5** showed comparable kinetics, with a sustained release of CO over time (Figure 2A). The kinetics of release were almost superimposable over the first 120 min, with negligible differences over the last 90 min. Despite slight differences in their *T*_1/10_ (Figure 2D), the comparison of the units of CO released over time (Table 2) displayed a very similar behavior within the series, suggesting that the substitution pattern on the aryl rings only slightly impacts their CO-releasing abilities. In particular, compounds **1**, **2** and **5**, bearing a sulfonyl group on the aryl ring at C5, released almost the same amount of CO at the end of the assay (0.64–0.68 CO units, Table 2). Compounds **3** and **4**, both decorated with a sulfamoylphenyl moiety at position 1 of the pyrrole, displayed very similar behavior and released a comparable amount of CO after 210 min (0.74–0.75 CO units, Table 2). Pyrrole **6** showed a completely different CO releasing profile (Figure 2B,C) and was the fastest and most efficient CO-releaser of the series, with a *T*_1/10_ value of 99.01 min (Figure 2D) and 0.92 CO units released after 210 min (Table 2). This compound showed a fast release of CO over the first 120 min, which reaches a maximum at 150 min and then slows down. When compared to its analogue **1**, compound **6** produced a 1.35-fold higher amount of Mb-CO at each time point until the end of the assay. These data suggest that the group bearing the DCH moiety and the chemical space around it strongly influences the CO release kinetic. The CO releasing behavior of compound **7** supported this hypothesis: indeed, it releases CO slower than compounds **1**-**5**, although reaching almost the same amount of Mb-CO after 210 min of incubation (Figure 2B,C). Differently from compounds **1**-**6**, the DCH portion of this compound is linked to a propynyl benzoate group at position 1 of the pyrrole ring, suggesting that the electronic structure can induce different CO releasing properties. Pyrazoles **8** and **9** showed different CO releasing profiles (Figure 2C). At the end of the assay, these compounds produced much smaller values of Mb-CO (1.75 μM and 1.22 μM for **8** and **9**, respectively, Table 1) than compounds **1**–**7** at the same time point. Therefore, it is interesting to note that the 3-((prop-2-yn-1-yloxy)-methyl)-1-pyrazole moiety is probably detrimental in terms of CO releasing efficiency when compared to acetate, carboxylate or benzoate moieties decorating compounds **1**–**7**. Previous studies reported the impact of the group bearing the DCH moiety in determining the CO releasing properties [22,23]. The different CO release kinetics observed for compounds **1**–**9** confirm the influence of the drug sphere on CO releasing properties and suggest that both the electronic density around the pyrrole/pyrazole ring and the group bearing the DCH moiety strongly impact the rate of CO release, yet further studies are needed to better characterize this phenomenon. To further explore the releasing properties of these derivatives, we selected compounds **1**, **5** and **6** to be studied at different CO-RM:Mb ratios (Figure 3).

As mentioned above, an excess of the CO acceptor (Mb) stimulates the CO release from CORMs. Thus, we expected a decrease in CO release at 1:1 CO-RM:Mb ratio. As shown in Figure 3, after 210 min of incubation, compounds **1**, **5** and **6** released a lower amount of CO when compared to the one observed in 1:6 conditions (Figure 2). Moreover, all the compounds released the same CO units (0.11–0.12 CO units), regardless of their different chemical structure. Therefore, disfavoring the CO release seems to reduce the differences in CO releasing efficiencies observed when Mb is present in excess (1:6/CO-RM:Mb).

### 3.3. Effects of Compounds ***1**–**5*** on Human Tenocytes

Once the influence of electronic and steric properties on CO release has been established, a fine-tuning of the drug sphere should also focus on treating particular conditions and on the biological activity of the drug sphere itself [17,34]. Compounds **1**-**5** drug sphere belongs to a series of sulphone and sulfamoyl diarylpyrrole derivatives developed by our research group as COX-2 selective inhibitors [35,36,37,38,39]. This class of compounds showed promising in vitro and in vivo anti-nociceptive and anti-inflammatory properties and tolerates a wide range of substituents at position C3. As augmented PGE_2_ levels are a marker of oxidative-stress inflammation, the modulation of PGE_2_ secretion might be a valuable strategy for therapeutic intervention of tendon diseases [27,40]. We therefore speculated that the conjugation of a COX-2 inhibiting scaffold and a CO-releasing moiety could help to achieve promising CORM-candidates for the treatment of tendon inflammatory-based diseases, as COX-2 inhibition and CO release could act synergically to resolve inflammation and restore oxidative homeostasis. Moreover, the conjugation of structural fragments of anti-inflammatory drugs with metal carbonyl moieties is well documented in the literature [18,19,24], and the five selected compounds showed quite similar CO release profiles (Figure 2), allowing us to make a proper comparison and rationalization of the observed biological activities. To help to discriminate between COX-2 mediated and independent activities, the efficacies of these compounds against inflammation and oxidative cytotoxicity were studied through the analysis of different parameters: the metabolic activity of tenocytes before and after H_2_O_2_ stimulation and the quantification of PGE_2_ secretion.

**Figure 4:**
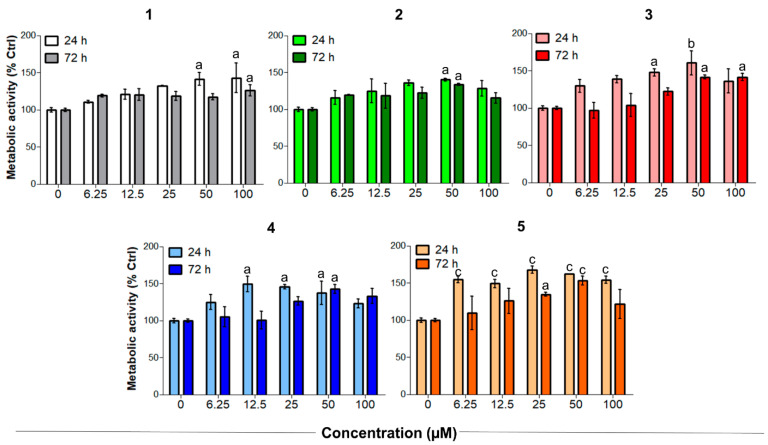
Metabolic activity of human primary tendon-derived cells exposed to increasing concentrations of CORMs (compounds **1**–**5**) after 24 and 72 h. The control sample (0 µM = cells treated with DMSO 0.1%) is set as 100%. a = *p* < 0.01; b = *p* < 0.001; c = *p* < 0.0001 between cells treated with CORMs and the control sample.

Tendinopathies are characterized by a higher level of tenocyte apoptosis and a decreased metabolic activity, which can reduce the resistance of tendon structures and lead to failure in healing [41,42]. Unstimulated tenocytes were therefore exposed to increasing concentrations of CORMs to evaluate their biocompatibility and effects under non-oxidative stress conditions (Figure 4). It is worth noting that Meloxicam exerted no significant effects on tenocytes when administered in the same experimental conditions [26].

On the other hand, compounds **1**–**5** significantly increased the metabolic activity of tendon-derived cells after 24 h, as observed for CAI-CORMs hybrids [26]. In more detail, all the tested compounds showed a dose-dependent rise up to 25–50 µM already after 24 h of exposure, which was particularly significant in the presence of compounds **3** and **5**. Interestingly, the tested compounds seemed less active after a 72 h exposure, as the metabolic activity was comparable to that of the control up to the concentration of 25 µM. This might be related to their slightly different CO release kinetics, but further studies are needed to corroborate this hypothesis.

### 3.4. Establishment of the Inflammatory Cell Model and Effects of Compounds ***1**–**5*** on Human Tenocytes under Oxidative Stress Conditions

**Figure 5:**
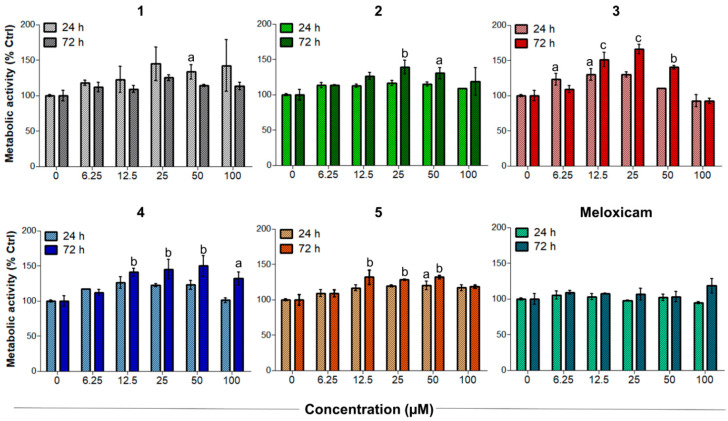
Metabolic activity of H_2_O_2_-pre-incubated human primary tendon-derived cells exposed to increasing concentrations of CORMs (compounds **1**–**5**) after 24 and 72 h. Cells were pre-incubated with H_2_O_2_ 100 µM for 3 h. The control sample (0 µM = cells pre-incubated with H_2_O_2_ and treated with DMSO 0.1%) is set as 100%. a = *p* < 0.01; b = *p* < 0.001; c = *p* < 0.0001 between cells treated with CORMs and the control sample.

These preliminary results highlighted that compounds **1**–**5** have good proliferative effects on tendon-derived cells and provided a proof-of-concept that the biological features of these compounds are not only COX-2 mediated but also rely on CO release. As reported elsewhere [43], increasing cell metabolism and proliferation are particularly important for tendon tissue repair after the acute inflammatory phase. With this rationale, compounds **1**–**5** were tested in sub-toxic oxidative stress conditions in vitro [26] to investigate their ability to counteract H_2_O_2_-induced oxidative stress. After 3 h of incubation with 100 µM H_2_O_2_, human tenocytes were exposed to increasing concentrations of CORMs. As reported in Figure 5, all the tested compounds were more active than Meloxicam in increasing the cell metabolism of tenocytes. Notably, compounds **3**–**5** were the most active of the series. In particular, compound **3** showed an outstanding efficacy, being able to increase metabolic activity up to 166.2% after 72 h when administered at 25 µM. Unlike under non-oxidative stress conditions, the percentage of metabolically active tenocytes increases after 72 h. This observation suggests a composite mechanism of action, which probably results from the combination of COX-2 inhibition and CO release. Consistent with previously obtained results, activities were maximum at 25 µM, then decreased at the higher concentrations tested.

### 3.5. Effects of Compound ***1**–**5*** on PGE_2_ Secretion

**Figure 6:**
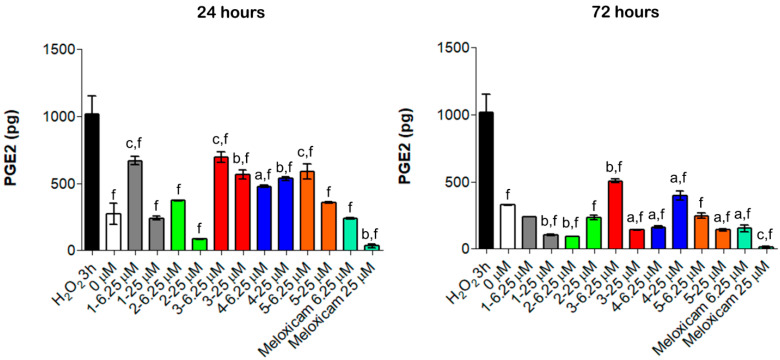
PGE_2_ secretion from H_2_O_2_-pre-incubated human primary tendon-derived cells in the presence of increasing concentrations of CORMs after 24 and 72 h. Cells were pre-incubated with H_2_O_2_ 100 µM for 3 h. 0 µM = cells pre-incubated with H_2_O_2_ and treated with DMSO 0.1%. The amount of PGE_2_ secreted (pg/mL) was normalized on cell metabolic activity data, resulting in the PGE_2_ secreted from each sample (total picograms). a = *p* < 0.01; b = *p* < 0.001; c = *p* < 0.0001 between cells treated with CORMs and the control sample. f = *p* < 0.0001 between cells exposed to CORMs and cells pre-treated with H_2_O_2_ for 3 h.

Anti-inflammatory COX-related activities of compounds **1**–**5** were evaluated by quantifying PGE_2_ secretion in the established oxidative stress conditions in vitro (Figure 6).

Consistent with literature data [27], even a short exposure to H_2_O_2_ for 3 h increased PGE_2_ secretion up to 1014.2 pg (Figure 6). Notably, all the tested compounds lowered the amount of PGE_2_ compared to the H_2_O_2_ pre-incubation but were less effective than Meloxicam (Figure 6). Compared to our previous experiments, the obtained data revealed a different trend of activity: compounds **1**, **2** and **5** showed remarkable anti-inflammatory effects and considerably reduced PGE_2_ secretion already at 24 h and mainly at 25 µM (240.5 pg, 84.8 pg and 357.1 pg, respectively), whereas compounds **3** and **4** were less efficient. Moreover, the modulation of PGE_2_ is time-dependent, being that this cytokine decreased over the time. Actually, the amount of PGE_2_ was almost halved with all the tested compounds after 72 h of exposure compared to 24 h treatment (Figure 6). These findings support the hypothesis that the observed anti-inflammatory effects rely on a COX-2 mediated mechanism of action and that the organometallic complexes retain the ability to inhibit COX-2.

Collectively, HO-1 expression and enzymatic activity are confirmed to influence positively and negatively both innate and adaptive immune responses; this dual action seems to be related to the stage of the inflammatory response or disease. The therapeutic potential of HO-1 may rely on limiting early inflammation, hampering successive tissue damage and modulating key pathways in most cell types of the immune system, given the complexity of heme catabolism and the role of HO-1 as a critical mediator of innate immune response. Immunomodulation is mostly related to higher demolition of the pro-inflammatory heme group, macrophage activation towards an anti-inflammatory macrophage profile with reduced secretion of pro-inflammatory cytokines and iNOS and interferon production by macrophages and dendritic cells. Indeed, T cells constitutively express HO-1, and their expansion regulatory is positively influenced by a tolerogenic phenotype sustained through HO-1 induction in dendritic cells. HO-1 modulation or application of low concentrations of CO to LPS-challenged macrophages reduced TNF-α and IL-1β expression and simultaneously stimulated the anti-inflammatory IL-10 production through p38-MAPK activity [44,45,46].

This context was particularly evident in models of tendon-related diseases. In this light, the role of the macrophage is an area of emerging interest in tendinopathies and in general in the healing of tendons. In fact, inflammation appears to be driven by a high number of infiltrating macrophages at the inflamed tendon site [47]. Furthermore, damaged tendons from patients with tendinopathy show an abundance of CD14^+^ and CD68^+^ activated macrophages [48]. We have already reported that CORM hybrids exert their biological effects both on inflamed macrophages and tenocytes [22,26], disclosing a challenging field of application for molecules active on the HO-1/CO molecular axis not strictly related to the immune system. Finally, although once considered cells not involved in the immune-regulation and only related to tendon remodeling, tenocytes have been disclosed as active cells, secreting cytokines and expressing inflammation-related proteins [27].

## 4. Conclusions

We developed a novel series of DCH-CORMs based on a 1,5-diarylpyrrole scaffold. The screening of three small subsets of 1,5-diarylpyrroles and pyrazoles in a CO release assay allowed us to define the influence of the drug sphere electronic density on the kinetics of CO release. Based on these results, a series of 1,5-diarylpyrroles containing structural fragments of COX-2 selective inhibitors were selected for further biological studies on human primary tendon-derived cells. The observed results suggested the existence of different mechanisms of action and allowed us to conclude that the activities of these compounds result from the combination of COX-inhibition and CO release. Indeed, the obtained data suggest a multiple role for compounds **1**–**5** in tendon-derived diseases: a direct effect on tendon homeostasis and a cytoprotective effect in human tenocytes exposed to oxidative stress, reflecting their activity as CO-releasers, and a reduction of PGE_2_ secretion, indicating a COX-2 mediated anti-inflammatory effect. Taken together, these findings indicate that these compounds could be potential double-acting therapeutic agents for the management of tendon-related diseases. Further studies are needed to better characterize their composite mechanism of action and the contribution of COX-2 inhibition to their biological activities.

## Figures and Tables

**Table 1:** MbCO formed at each time point when compounds **1**–**9** were analyzed at 1:6 CORM:Mb ratio.

Time (min)	MbCO Formed (μM)								
	1	2	3	4	5	6	7	8	9
0	0	0	0	0	0	0	0	0	0
30	0.56	0.58	0.50	0.52	0.63	0.82	0.57	0.56	0.08
60	0.88	1.03	1.01	1.15	1.09	1.46	0.91	0.77	0.45
90	1.49	1.59	1.50	1.42	1.35	2.09	1.27	0.95	0.59
120	1.90	1.76	1.90	1.86	1.57	2.45	1.52	1.24	0.74
150	2.32	2.06	2.07	2.37	1.72	3.12	1.68	1.37	0.99
180	2.28	2.20	2.31	2.35	1.85	3.08	1.88	1.57	1.09
210	2.25	2.22	2.48	2.51	2.12	3.05	2.00	1.75	1.22

**Table 2:** CO units released by compounds **1**–**9** after 210 min of incubation working at 1:6 CORM-Mb ratio and by compounds **1**, **5** and **6** at 1:1 CORM-Mb ratio.

CO Units Released after 210 min	1	2	3	4	5	6	7	8	9
3.33 μM (1:6)	0.68	0.67	0.74	0.75	0.64	0.92	0.60	0.53	0.37
20 μM (1:1)	0.11	-	-	-	0.11	0.12	-	-	-

## Data Availability

Data are contained within the article.

## Supplementary Materials

The following are available online at https://www.mdpi.com/article/10.3390/antiox10111828/s1, Scheme S1: Synthetic pathway for compounds **1**–**5**, Scheme S2: Synthetic pathway for compound **6**, Scheme S3: Synthetic pathway for compound **7**, Scheme S4: Synthetic pathway for compounds **8**,**9**.

## References

[1] Zobi F. (2013). CO and CO-Releasing Molecules in Medicinal Chemistry. Future Med. Chem..

[2] Ryter S.W., Choi A.M.K. (2009). Heme Oxygenase-1/Carbon Monoxide. Am. J. Respir. Cell. Mol. Biol..

[3] Constantin M., Choi A.J.S., Cloonan S.M., Ryter S.W. (2012). Therapeutic Potential of Heme Oxygenase-1/Carbon Monoxide in Lung Disease. Int. J. Hypertens.

[4] Motterlini R., Haas B., Foresti R. (2012). Emerging Concepts on the Anti-Inflammatory Actions of Carbon Monoxide-Releasing Molecules (CO-RMs). Med. Gas. Res..

[5] Castruccio Castracani C., Longhitano L., Distefano A., Di Rosa M., Pittalà V., Lupo G., Caruso M., Corona D., Tibullo D., Li Volti G. (2020). Heme Oxygenase-1 and Carbon Monoxide Regulate Growth and Progression in Glioblastoma Cells. Mol. Neurobiol..

[6] Di Pietro C., Öz H.H., Murray T.S., Bruscia E.M. (2020). Targeting the Heme Oxygenase 1/Carbon Monoxide Pathway to Resolve Lung Hyper-Inflammation and Restore a Regulated Immune Response in Cystic Fibrosis. Front. Pharmacol..

[7] Pol O. (2021). The Role of Carbon Monoxide, Heme Oxygenase 1, and the Nrf2 Transcription Factor in the Modulation of Chronic Pain and Their Interactions with Opioids and Cannabinoids. Med. Res. Rev..

[8] Motterlini R., Otterbein L.E. (2010). The Therapeutic Potential of Carbon Monoxide. Nat. Rev. Drug. Discov..

[9] Foresti R., Bani-Hani M.G., Motterlini R. (2008). Use of Carbon Monoxide as a Therapeutic Agent: Promises and Challenges. Intensive Care Med..

[10] Knauert M., Vangala S., Haslip M., Lee P.J. (2013). Therapeutic Applications of Carbon Monoxide. Oxid. Med. Cell. Longev..

[11] Hess D.R. (2017). Inhaled Carbon Monoxide: From Toxin to Therapy. Respir. Care.

[12] Adach W., Błaszczyk M., Olas B. (2020). Carbon Monoxide and Its Donors—Chemical and Biological Properties. Chem. Biol. Interact.

[13] Goebel U., Wollborn J. (2020). Carbon Monoxide in Intensive Care Medicine—Time to Start the Therapeutic Application?!. Intensive Care Med. Exp..

[14] Ling K., Men F., Wang W.-C., Zhou Y.-Q., Zhang H.-W., Ye D.-W. (2018). Carbon Monoxide and Its Controlled Release: Therapeutic Application, Detection, and Development of Carbon Monoxide Releasing Molecules (CORMs). J. Med. Chem..

[15] Cavicchioli F., Cesarotti I.M., Fangman M., Lua J., Hautamaki R., Doré S. (2021). Carbon Monoxide Therapy Using Hybrid Carbon Monoxide-Releasing/Nrf2-Inducing Molecules through a Neuroprotective Lens. Chemistry.

[16] Kautz A.C., Kunz P.C., Janiak C. (2016). CO-Releasing Molecule (CORM) Conjugate Systems. Dalton Trans..

[17] Romão C.C., Blättler W.A., Seixas J.D., Bernardes G.J.L. (2012). Developing Drug Molecules for Therapy with Carbon Monoxide. Chem. Soc. Rev..

[18] Ott I., Kircher B., Bagowski C.P., Vlecken D.H.W., Ott E.B., Will J., Bensdorf K., Sheldrick W.S., Gust R. (2009). Modulation of the Biological Properties of Aspirin by Formation of a Bioorganometallic Derivative. Angew. Chem. Int. Ed. Engl..

[19] Zanellato I., Bonarrigo I., Ravera M., Gabano E., Gust R., Osella D. (2013). The Hexacarbonyldicobalt Derivative of Aspirin Acts as a CO-Releasing NSAID on Malignant Mesothelioma Cells. Metallomics.

[20] Heffern M.C., Yamamoto N., Holbrook R.J., Eckermann A.L., Meade T.J. (2013). Cobalt Derivatives as Promising Therapeutic Agents. Curr. Opin. Chem. Biol..

[21] Gong Y., Zhang T., Liu H., Zheng Y., Li N., Zhao Q., Chen Y., Liu B. (2015). Synthesis, Toxicities and Cell Proliferation Inhibition of CO-Releasing Molecules Containing Cobalt. Transit. Met. Chem..

[22] Berrino E., Carradori S., Angeli A., Carta F., Supuran C.T., Guglielmi P., Coletti C., Paciotti R., Schweikl H., Maestrelli F. (2021). Dual Carbonic Anhydrase IX/XII Inhibitors and Carbon Monoxide Releasing Molecules Modulate LPS-Mediated Inflammation in Mouse Macrophages. Antioxidants.

[23] Berrino E., Milazzo L., Micheli L., Vullo D., Angeli A., Bozdag M., Nocentini A., Menicatti M., Bartolucci G., di Cesare Mannelli L. (2019). Synthesis and Evaluation of Carbonic Anhydrase Inhibitors with Carbon Monoxide Releasing Properties for the Management of Rheumatoid Arthritis. J. Med. Chem..

[24] Li J., Zhang J., Zhang Q., Bai Z., Zhao Q., He D., Wang Z., Chen Y., Liu B. (2018). Syntheses and Anti-Cancer Activity of CO-Releasing Molecules with Targeting Galactose Receptors. Org. Biomol. Chem..

[25] Perontsis S., Dimitriou A., Fotiadou P., Hatzidimitriou A.G., Papadopoulos A.N., Psomas G. (2019). Cobalt(II) Complexes with the Non-Steroidal Anti-Inflammatory Drug Diclofenac and Nitrogen-Donor Ligands. J. Inorg. Biochem..

[26] Gallorini M., Berardi A.C., Ricci A., Antonetti Lamorgese Passeri C., Zara S., Oliva F., Cataldi A., Carta F., Carradori S. (2021). Dual Acting Carbon Monoxide Releasing Molecules and Carbonic Anhydrase Inhibitors Differentially Modulate Inflammation in Human Tenocytes. Biomedicines.

[27] Oliva F., Gallorini M., Antonetti Lamorgese Passeri C., Gissi C., Ricci A., Cataldi A., Colosimo A., Berardi A.C. (2020). Conjugation with Methylsulfonylmethane Improves Hyaluronic Acid Anti-Inflammatory Activity in a Hydrogen Peroxide-Exposed Tenocyte Culture In Vitro Model. Int. J. Mol. Sci..

[28] Darrieutort-Laffite C., Soslowsky L.J., Le Goff B. (2020). Molecular and Structural Effects of Percutaneous Interventions in Chronic Achilles Tendinopathy. Int. J. Mol. Sci..

[29] Zara S., De Colli M., di Giacomo V., Zizzari V.L., Di Nisio C., Di Tore U., Salini V., Gallorini M., Tetè S., Cataldi A. (2015). Zoledronic acid at subtoxic dose extends osteoblastic stage span of primary human osteoblasts. Clin. Oral. Investig..

[30] Marconi G.D., Gallorini M., Carradori S., Guglielmi P., Cataldi A., Zara S. (2019). The Up-Regulation of Oxidative Stress as a Potential Mechanism of Novel MAO-B Inhibitors for Glioblastoma Treatment. Molecules.

[31] Atkin A.J., Lynam J.M., Moulton B.E., Sawle P., Motterlini R., Boyle N.M., Pryce M.T., Fairlamb I.J.S. (2011). Modification of the Deoxy-Myoglobin/Carbonmonoxy-Myoglobin UV-Vis Assay for Reliable Determination of CO-Release Rates from Organometallic Carbonyl Complexes. Dalton Trans..

[32] Smulevich G., Droghetti E., Focardi C., Coletta M., Ciaccio C., Nocentini M. (2007). A Rapid Spectroscopic Method to Detect the Fraudulent Treatment of Tuna Fish with Carbon Monoxide. Food Chem..

[33] Wilson J.L., Fayad Kobeissi S., Oudir S., Haas B., Michel B., Dubois Randé J.-L., Ollivier A., Martens T., Rivard M., Motterlini R. (2014). Design and Synthesis of New Hybrid Molecules That Activate the Transcription Factor Nrf2 and Simultaneously Release Carbon Monoxide. Chem. Eur. J..

[34] García-Gallego S., Bernardes G.J.L. (2014). Carbon-Monoxide-Releasing Molecules for the Delivery of Therapeutic CO in Vivo. Angew. Chem. Int. Ed. Engl..

[35] Biava M., Porretta G.C., Poce G., Supino S., Forli S., Rovini M., Cappelli A., Manetti F., Botta M., Sautebin L. (2007). Cyclooxygenase-2 Inhibitors. 1,5-Diarylpyrrol-3-Acetic Esters with Enhanced Inhibitory Activity toward Cyclooxygenase-2 and Improved Cyclooxygenase-2/Cyclooxygenase-1 Selectivity. J. Med. Chem..

[36] Biava M., Porretta G.C., Poce G., Supino S., Manetti F., Forli S., Botta M., Sautebin L., Rossi A., Pergola C. (2008). Synthesis, in Vitro, and in Vivo Biological Evaluation and Molecular Docking Simulations of Chiral Alcohol and Ether Derivatives of the 1,5-Diarylpyrrole Scaffold as Novel Anti-Inflammatory and Analgesic Agents. Bioorg. Med. Chem..

[37] Biava M., Porretta G.C., Poce G., Battilocchio C., Manetti F., Botta M., Forli S., Sautebin L., Rossi A., Pergola C. (2010). Novel Ester and Acid Derivatives of the 1,5-Diarylpyrrole Scaffold as Anti-Inflammatory and Analgesic Agents. Synthesis and in Vitro and in Vivo Biological Evaluation. J. Med. Chem..

[38] Battilocchio C., Poce G., Alfonso S., Porretta G.C., Consalvi S., Sautebin L., Pace S., Rossi A., Ghelardini C., Di Cesare Mannelli L. (2013). A Class of Pyrrole Derivatives Endowed with Analgesic/Anti-Inflammatory Activity. Bioorg. Med. Chem..

[39] Consalvi S., Alfonso S., Di Capua A., Poce G., Pirolli A., Sabatino M., Ragno R., Anzini M., Sartini S., La Motta C. (2015). Synthesis, Biological Evaluation and Docking Analysis of a New Series of Methylsulfonyl and Sulfamoyl Acetamides and Ethyl Acetates as Potent COX-2 Inhibitors. Bioorg. Med. Chem..

[40] Bergqvist F., Carr A.J., Wheway K., Watkins B., Oppermann U., Jakobsson P.-J., Dakin S.G. (2019). Divergent Roles of Prostacyclin and PGE2 in Human Tendinopathy. Arthritis Res. Ther..

[41] Osti L., Berardocco M., di Giacomo V., Di Bernardo G., Oliva F., Berardi A.C. (2015). Hyaluronic Acid Increases Tendon Derived Cell Viability and Collagen Type I Expression in Vitro: Comparative Study of Four Different Hyaluronic Acid Preparations by Molecular Weight. BMC Musculoskelet Disord..

[42] Gallorini M., Petzel C., Bolay C., Hiller K.-A., Cataldi A., Buchalla W., Krifka S., Schweikl H. (2015). Activation of the Nrf2-Regulated Antioxidant Cell Response Inhibits HEMA-Induced Oxidative Stress and Supports Cell Viability. Biomaterials.

[43] Sharma P., Maffulli N. (2006). Biology of Tendon Injury: Healing, Modeling and Remodeling. J. Musculoskelet. Neuronal. Interact..

[44] Canesin G., Hejazi S.M., Swanson K.D., Wegiel B. (2020). Heme-Derived Metabolic Signals Dictate Immune Responses. Front. Immunol..

[45] Fernández-Fierro A., Funes S.C., Rios M., Covián C., González J., Kalergis A.M. (2020). Immune Modulation by Inhibitors of the HO System. Int. J. Mol. Sci..

[46] Campbell N.K., Fitzgerald H.K., Dunne A. (2021). Regulation of Inflammation by the Antioxidant Haem Oxygenase 1. Nat. Rev. Immunol..

[47] Frich L.H., Fernandes L.R., Schrøder H.D., Hejbøl E.K., Nielsen P.V., Jørgensen P.H., Stensballe A., Lambertsen K.L. (2021). The Inflammatory Response of the Supraspinatus Muscle in Rotator Cuff Tear Conditions. J. Shoulder. Elbow. Surg..

[48] Dakin S.G., Newton J., Martinez F.O., Hedley R., Gwilym S., Jones N., Reid H.A.B., Wood S., Wells G., Appleton L. (2018). Chronic Inflammation Is a Feature of Achilles Tendinopathy and Rupture. Br. J. Sports. Med..

