# Supplementary material for: A Novel Class of Dual-Acting DCH-CORMs Counteracts Oxidative Stress-Induced Inflammation in Human Primary Tenocytes
Source: Antioxidants (Basel). 2021 Nov 18;10(11):1828. doi: 10.3390/antiox10111828 (PMC8614895; doi:10.3390/antiox10111828)
Supplement: Supplementary file 1 [file antioxidants-10-01828-s001.zip › antioxidants-1442147-supplementary.pdf]

Supporting information

A novel class of dual-acting DCH-CORMs counteracts oxidative stress-induced inflammation in human primary tenocytes.

Federico Appetecchia[1†], Sara Consalvi[1†], Emanuela Berrino[1], Marialucia Gallorini[2], Arianna Granese[1], Cristina Campestre[2], Simone Carradori[2*], Mariangela Biava[1*] and Giovanna Poce[1*]

[1]Department of Chemistry and Technologies of Drug, Sapienza University of Rome, piazzale A. Moro 5, 00185 Rome, Italy.

[2]Department of Pharmacy, "G. d'Annunzio" University of Chieti-Pescara, via dei Vestini 31, 66100 Chieti, Italy.

[†]F. Appetecchia and S. Consalvi contributed equally to the work.

*****Corresponding Authors. E-mail address: simone.carradori@unich.it (S. Carradori); mariangela.biava@uniroma1.it (M. Biava); giovanna.poce@uniroma1.it (G. Poce)

Table of contents:

Chemistry ____ S3

General procedures ____ S7

Full experimental details and ^{1}H NMR for all intermediates ____ S10

Full experimental details and ^{1}H NMR and ^{13}C NMR for final compounds **1-9** ____ S13

References ____ S16

Chemistry

All chemicals used were obtained from commercial sources (Merck, Acros, Syngene) and were used as supplied without further purification. Compound **17** was commercially available and purchased from Syngene. Merck silica gel 60 (230–400 mesh) and Merck aluminum oxide (activity II-III, according to Brockmann) were used for chromatographic purifications with the indicated solvents. All operations were monitored by Merck TLC plates (silica gel 60 F 254 and Aluminium oxide F254) and then compounds were visualized under UV light (254 and 365 nm) and/or stained with the relevant reagent. The yields refer to the purified products, and they were not optimized. ^{13}C NMR and ^{1}H NMR spectra were recorded on a Bruker Avance III NMR 400 spectrometer in the indicated solvent with reference to tetramethylsilane (TMS). The values of the chemical shifts are expressed in parts per million (ppm) and the coupling constants (*J*) in hertz with signal multiplicities reported as singlet (s), doublet (d), triplet (t), quadruplet (q) and multiplet (m). When specified, systematic compound names were generated by ChemDraw Professional 16.0 following IUPAC conventions.

Compounds **1-5** were synthesized as reported in Scheme 1. Briefly, 1,5-diarylpyrroles **11a-d** were obtained through a reaction between 1,4-pentandiones **10a-c** and the suitable aniline according to Paal-Knorr conditions. Sulfamoylpyrrole **11e** was obtained as previously reported [1]. Pyrroles **11a-e** were then regioselectively acylated with ethoxalyl chloride and $TiCl_4$, affording ketoesters **12a-e**. The latter were reduced with triethylsilane and trifluoroacetic acid (TFA) to the ethyl esters **13a-e**, which were then hydrolized with NaOH, providing acids **14a-e** in very good yields. Propargylic derivatives **15a-b** were synthesized by reacting acids **14a-b** and propargyl bromide, using potassium carbonate as a base. To prevent side reactions, derivatives bearing a sulfamoyl moiety (**15c-e**) were synthesized by coupling acids **14c-e** and propargyl alcohol, using EDCI as activating agent and DMAP as covalent nucleophilic catalyst. The terminal alkyne of propargylic intermediates was then reacted with hexacarbonyl dicobalt providing compounds **1-5** in good yields.

Scheme S1: synthetic pathway for compounds **1-5**.

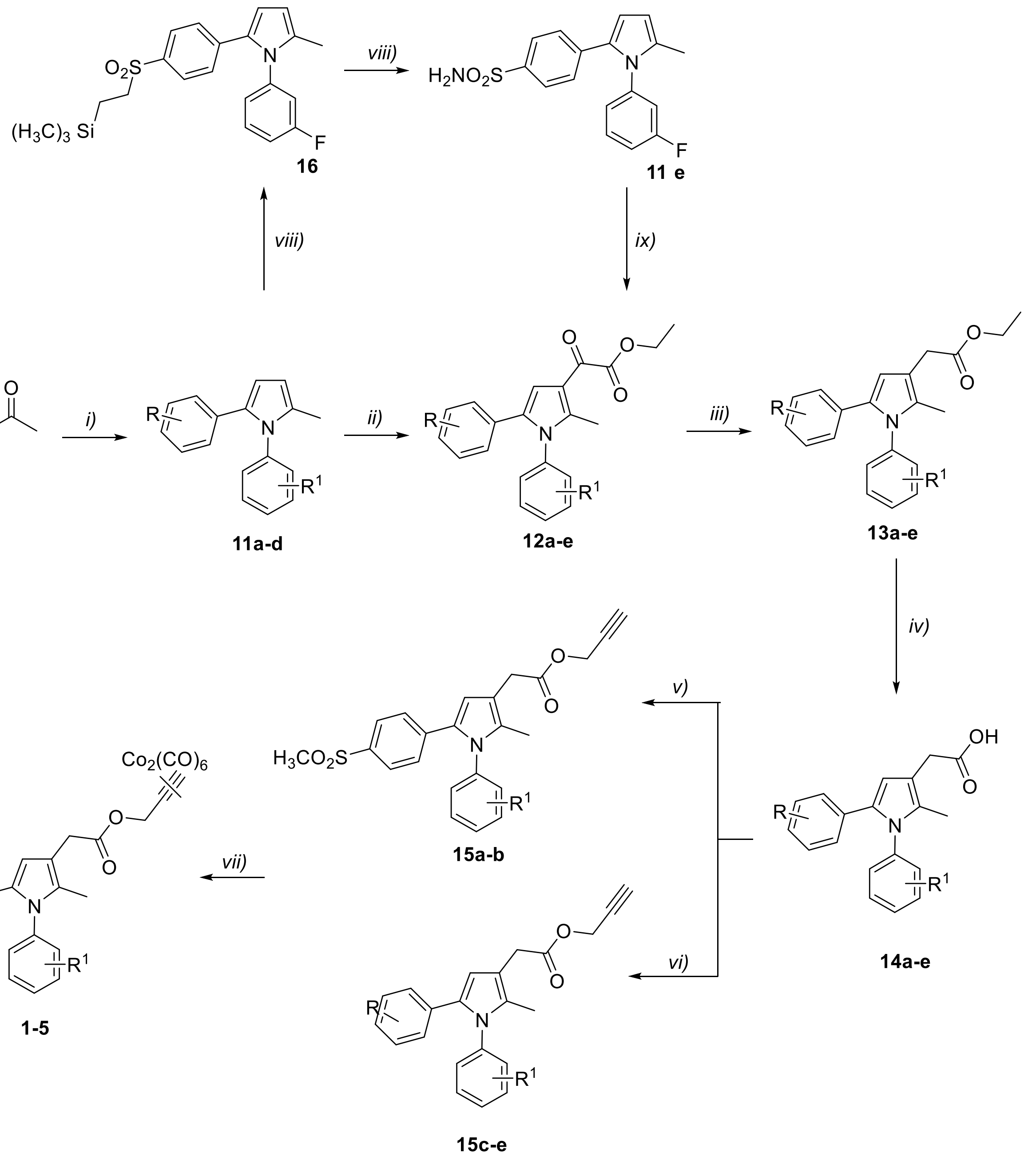

Reagents and conditions: *i)* R^1PhNH_2, PTSA, EtOH, reflux, 5h; *ii)* $CH_3CH_2OCOCOCl$, $TiCl_4$, CH_2Cl_2, room temperature, 4 h; *iii)* Et_3SiH, CF_3COOH, room temperature, 3h; *iv)* NaOH aq. 1N, EtOH, reflux, 1h; *v)* $BrCH_2CCH$, K_2CO_3, DMF, 40°C, 1h; *vi)* $OHCH_2CCH$, NEt_3, DMAP, EDCI, room temperature, 24h; *vii)* $Co_2(CO)_8$, THF, room temperature, 2h; *viii)* *n*-BuLi, $(Me_2CH)_2NH$, THF, -78°C, 1.5h, then $(CH_3)_3CH_2SiI$,room temperature, 24h; *ix)* Bu_4NF, THF, reflux, 1.5h, then NH_2OSO_3H, CH_3COONa, room temperature**,** 1h.

Derivative **6** was easily synthesized according to the synthetic pathway reported in Scheme 2: a reaction between the commercially available acid **17** and propargylic bromide afforded the propargylic ester **18**, which was then reacted with hexacarbonyl dicobalt to yield the DCH complex **6**.

Scheme S2: synthetic pathway for compound **6**.

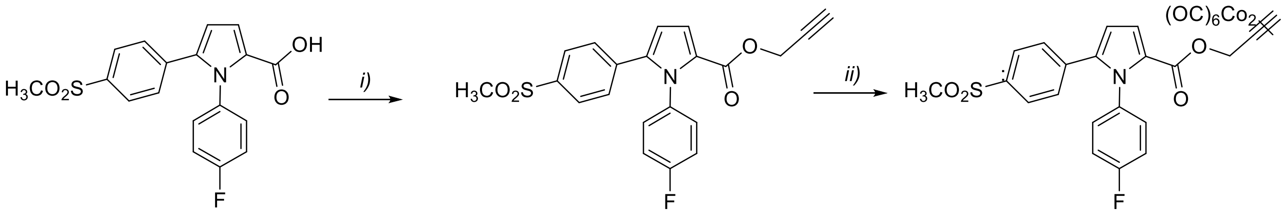

17 **18** **6**

Reagents and conditions: *i)* $BrCH_2CCH$, K_2CO_3, DMF, 40°C, 1h; *ii)* $Co_2(CO)_8$, THF, room temperature, 2h.

Derivative **7** was prepared as shown in Scheme S3. A Paal-Knorr condensation of 1,4-pentandione **10c** and *p*-aminobenzoic acid gave pyrrole **19** which was in turn reacted with propargyl bromide in the presence of potassium carbonate. The so obtained propargylic ester **20** underwent a reaction with octacarbonyl dicobalt, affording final compound **7** in 70% yield.

Scheme S3: synthetic pathway for compound **7**.

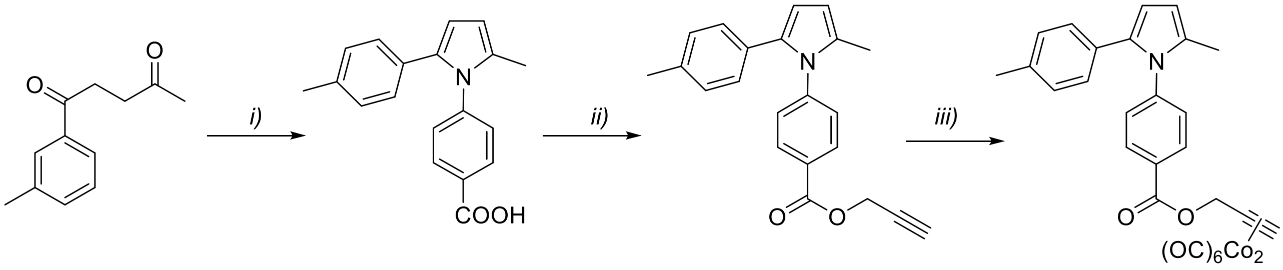

10c **19** **20** **7**

Reagents and conditions: *i)* 4-$NH_2PhCOOH$, PTSA, EtOH, reflux, 5h; *ii)* $BrCH_2CCH$, K_2CO_3, DMF, 40°C, 1h; *iii)* $Co_2(CO)_8$, THF, room temperature, 2h.

1,5-diarylpyrazole alcohols **25a-b** were obtained according to a previously reported procedure [2] and then reacted with propargyl bromide using sodium hydride as a base. The so obtained propargylic ethers **26a-b** were then treated with hexacarbonyl dicobalt affording compounds **8-9** in satisfactory yields (Scheme S4).

Scheme S4: synthetic pathway for compounds **8-9**.

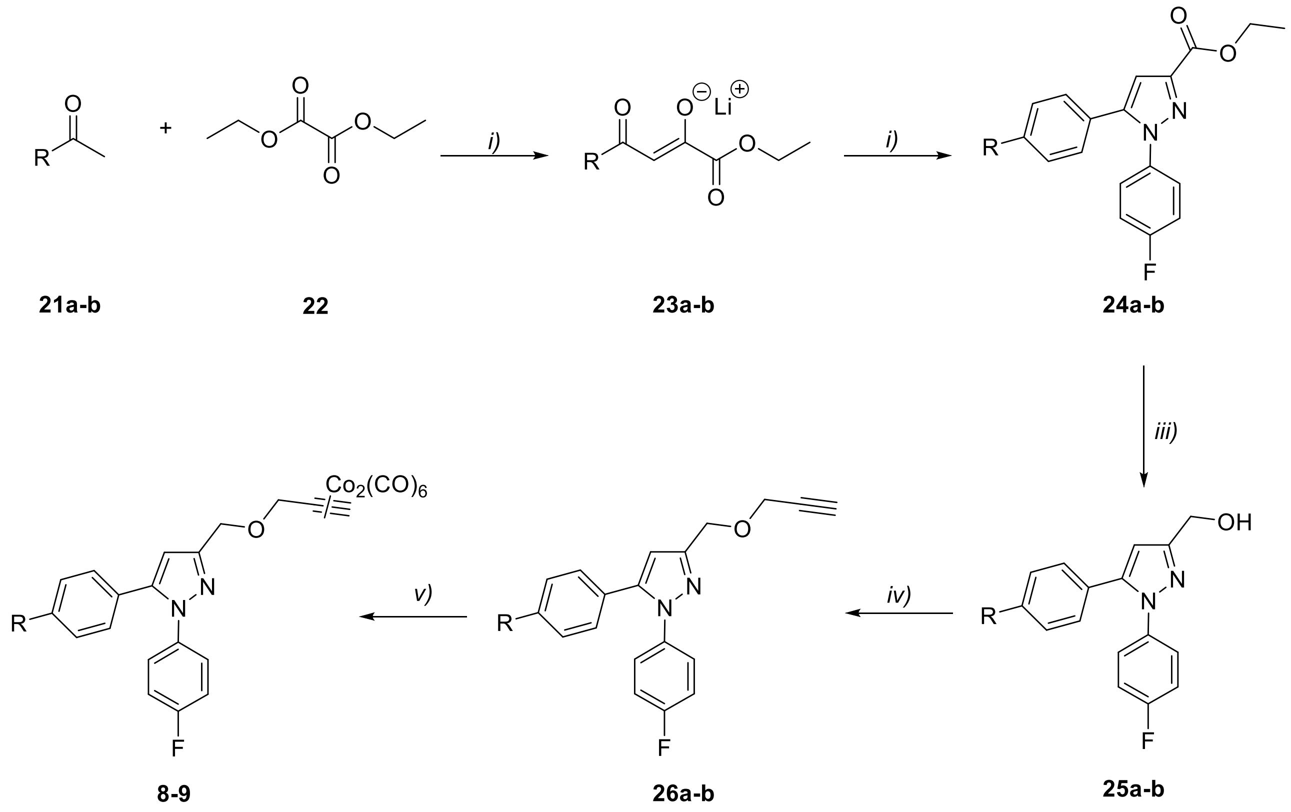

Reagents and conditions: *i)* $LiN(Si(CH_3)_3)_2$, THF, -78 °C and then room temperature, 24 h; *ii)* $FC_6H_4NHNH_2$.HCl, EtOH, reflux, 5h; *iii)* $LiAlH_4$, THF, 0°C, 1h, room temperature, 3h; *iv)* $BrCH_2CCH$, NaH, THF, room temperature, 2h; *v)* $Co_2(CO)_8$, THF, room temperature, 2h.

General procedures

General procedure for the preparation of 1,5-diarylpyrroles 11a-d and 19.

Compounds **11a-d** were obtained according to Paal-Knorr condensation conditions. 1,4-pentandiones **10a-c** (2.28 mmol), obtained as previously reported [3,4], were dissolved in ethanol (50 mL), then the suitable aniline (2.50 mmol) and *p*-toluenesulfonic acid (0.17 mmol) were added in sequence. The mixture was refluxed for 3 h and was then cooled down and concentrated under reduced pressure. Thereafter, the crude material was purified on silica gel using a mixture of petroleum ether/ethyl acetate 3:1 (v/v) (compounds **11a-b**) or dichloromethane (DCM)/methanol 2% (v/v) (compounds **11c-d**), giving the desired products in very good yields (60-83%).

1-(3-fluorophenyl)-2-methyl-5-(4-(methylsulfonyl)phenyl)-1H-pyrrole (11a). Physicochemical, spectroscopic, and analytical data are consistent with those reported in the literature [4].

1-(4-fluorophenyl)-2-methyl-5-(4-(methylsulfonyl)phenyl)-1H-pyrrole (11b). Physicochemical, spectroscopic, and analytical data are consistent with those reported in the literature [4].

4-(2-(3,4-difluorophenyl)-5-methyl-1*H*-pyrrol-1-yl)benzenesulfonamide (11c). White powder, 80% yield. ^{1}H NMR (400 MHz, $CDCl_3$): δ ppm= 7.70 (d, 2H, *J*= 8.5 Hz), 7.30-7.26 (m, 1 H), 7.18 (d, 2H, *J*= 8.5 Hz), 7.06-7.02 (m, 1H), 6.97-6.93 (m, 1H), 6.40 (d, 1H, *J* = 3.4 Hz), 6.19 (d, 1H, *J* = 3.4 Hz), 4.92 (s broad, 2H), 2.10 (s, 3H).

4-(2-methyl-5-(*p*-tolyl)-1*H*-pyrrol-1-yl)benzenesulfonamide (11d). White powder, 83% yield. ^{1}H NMR (400 MHz, $CDCl_3$): δ ppm= 7.91 (d, 2H, *J* = 8.4 Hz), 7.27 (d, 2H, *J* = 8.4 Hz), 6.97 (d, 2H, *J* = 8.0 Hz), 6.91 (d, 2H, *J* = 8.0 Hz), 6.32 (d, 1H, *J* = 3.3 Hz), 6.12 (d, 1H, *J* = 3.3 Hz), 4.92 (s broad, 2H), 2.27 (s, 3H), 2.16 (s, 3H).

4-(2-methyl-5-(*p*-tolyl)-1*H*-pyrrol-1-yl)benzoic acid (19): white powder, 60% yield. ^{1}H NMR ($CDCl_3$) δ ppm: 8.21 (d, 2H, *J*= 8.3 Hz), 7.63 (d, 2H, *J*= 8.3 Hz), 7.13 (d, 2H, *J*= 8.1 Hz), 7.07 (d, 2H, *J*= 8.1 Hz), 6.32 (d, 1H, *J*= 3.3 Hz), 6.20 (d, 1H, *J*= 3.3 Hz), 2.34 (s, 3H), 2.20 (s, 3H).

General procedure for the preparation of 1,5-diarylpyrrole-3-glyoxylic esters 12a-e.

To a solution of the appropriate pyrrole (**11a-e**) (9 mmol) in anhydrous DCM (20 mL), ethoxalyl chloride (0.38 mL) and $TiCl_4$ (0.34 mL) were added at 0 °C under a nitrogen flow. The resulting purple mixture was left to react at room temperature for 4 h and was then diluted with water, stirred for an additional 30 minutes, and extracted with DCM. The organic layers were washed with brine and dried over Na_2SO_4. After filtration and removal of the solvent under vacuum, the crude residue was purified by column chromatography on silica gel using a mixture of petroleum ether/ethyl acetate (3:1 (v/v)) to afford compounds **12a-e** as pale-yellow solids.

Ethyl 2-(1-(3-fluorophenyl)-2-methyl-5-(4-(methylsulfonyl)phenyl)-1H-pyrrol-3-yl)-2-oxoacetate (12a). Physicochemical, spectroscopic, and analytical data are consistent with those reported in the literature [4].

Ethyl 2-(1-(4-fluorophenyl)-2-methyl-5-(4-(methylsulfonyl)phenyl)-1H-pyrrol-3-yl)-2-oxoacetate (12b). Physicochemical, spectroscopic, and analytical data are consistent with those reported in the literature [4].

Ethyl 2-(5-(3,4-difluorophenyl)-2-methyl-1-(4-sulfamoylphenyl)-1*H*-pyrrol-3-yl)-2-oxoacetate (**12c**). Pale-yellow powder, 50% yield. ^{1}H NMR (400 MHz, $CDCl_3$): δ ppm= 7.78 (d, 2H, *J*= 8.5 Hz), 7.32-7.27 (m, 1 H), 7.24 (d, 2H, *J*= 8.5 Hz), 7.10 (s, 1H), 7.06-7.02 (m, 1H), 6.97-6.93 (m, 1H), 4.43 (q, 1H, *J*= 7.1 Hz), 3.04 (s, 3H), 2.47 (s, 3H), 1.43 (t, 2H, *J*= 7.1 Hz).

Ethyl 2-(2-methyl-1-(4-sulfamoylphenyl)-5-(*p*-tolyl)-1*H*-pyrrol-3-yl)-2-oxoacetate (**12d**). Pale-yellow powder, 55% yield. ^{1}H NMR (400 MHz, $CDCl_3$): δ ppm= 7.97 (d, 2H, *J*= 8.5 Hz), 7.28 (d, 2H, *J*= 8.5 Hz), 6.99 (d, 2H, *J*= 8.0Hz), 6.91-6.88 (m, 3H), 4.92 (s broad, 2H), 4.42 (q, 2H, *J*= 7.1Hz), 2.47 (s, 3H), 2.28 (s, 3H), 1.43 (t, 3H, *J*= 7.1 Hz).

Ethyl 2-(1-(3-fluorophenyl)-2-methyl-5-(4-sulfamoylphenyl)-1H-pyrrol-3-yl)-2-oxoacetate (12e). Physicochemical, spectroscopic, and analytical data are consistent with those reported in the literature [1].

General procedure for the synthesis of ethyl 1,5-diarylpyrrole-3-acetic esters 13a-e.

To a solution of the suitable glyoxylic derivative (**12a-e**) (2.3 mmol) in TFA (9 mL), triethylsilane (0.75 mL) was slowly added at 0 °C and under a nitrogen atmosphere. The mixture was stirred for 2 h at room temperature and then the reaction was quenched with 40% aqueous ammonia (10 mL) and extracted with DCM. The organic layers were then washed with brine, dried over Na_2SO_4, filtered, and concentrated under vacuum. The resulting residue was purified on silica gel using a mixture of petroleum ether/ethyl acetate (2:1 (v/v)) as the mobile phase and giving compounds **13a-e** as yellowish solids.

Ethyl 2-(1-(3-fluorophenyl)-2-methyl-5-(4-(methylsulfonyl)phenyl)-1H-pyrrol-3-yl)acetate (13a) Physicochemical, spectroscopic, and analytical data are consistent with those reported in the literature [4].

Ethyl 2-(1-(4-fluorophenyl)-2-methyl-5-(4-(methylsulfonyl)phenyl)-1H-pyrrol-3-yl)acetate (13b) Physicochemical, spectroscopic, and analytical data are consistent with those reported in the literature [4].

Ethyl 2-(5-(3,4-difluorophenyl)-2-methyl-1-(4-sulfamoylphenyl)-1*H*-pyrrol-3-yl)acetate (**13c**). Pale-yellow powder, 40% yield. ^{1}H NMR (400 MHz, DMSO-d_6): δ ppm= 7.87 (d, 2H, *J*= 8.5 Hz), 7.50 (s broad, 2H), 7.41 (d, 2H, *J*= 8.5 Hz), 7.30-7.23 (m, 1H), 7.05-7.00 (m, 1H), 6.80-6.77 (m, 1H), 6.39 (s, 1H), 4.10 (q, 2H, *J* = 7.1 Hz), 3.49 (s, 2H), 2.01 (s, 3H), 1.21 (t, 2H, *J* = 7.1 Hz).

Ethyl 2-(2-methyl-1-(4-sulfamoylphenyl)-5-(*p*-tolyl)-1*H*-pyrrol-3-yl)acetate (**13d**). Pale-yellow powder, 45% yield. ^{1}H NMR (400 MHz, $CDCl_3$) δ ppm: 7.90 (d, 2H, *J*= 8.6 Hz), 7.25 (d, 2H, *J*= 8.6 Hz), 6.96 (d, 2H, *J*= 8.1 Hz), 6.89 (d, 2H, *J*= 8.1 Hz), 6.33 (s, 1H), 4.86 (s broad, 2H), 4.19 (q, 2H, *J* = 7.1 Hz), 3.50 (s, 2H), 2.26 (s, 3H), 2.10 (s, 3H), 1.30 (t, 2H, *J* = 7.1 Hz).

Ethyl 2-(1-(4-fluorophenyl)-2-methyl-5-(4-(sulfamoylphenyl)-1H-pyrrol-3-yl)acetate (13e). Physicochemical, spectroscopic, and analytical data are consistent with those reported in the literature [1].

General procedure for the preparation of 1,5-diarylpyrrole-3-acetic acids 14a-e.

The appropriate 1,5-diarylpyrrole-3-acetic ester (**13a-e**) (2.2 mmol) was dissolved in ethanol (15 mL) and 1N NaOH (15 mL) was slowly added to the solution. The mixture was refluxed for 2 h and then cooled down to room temperature and concentrated under vacuum. The residue was dissolved in water and acidified with 37% HCl. The so obtained precipitate was filtered off affording the acids **14a-e** in very good yields (75-90%).

2-(1-(3-fluorophenyl)-2-methyl-5-(4-(methylsulfonyl)phenyl)-1H-pyrrol-3-yl)acetic acid (14a). Physicochemical, spectroscopic, and analytical data are consistent with those reported in the literature [5].

2-(1-(4-fluorophenyl)-2-methyl-5-(4-(methylsulfonyl)phenyl)-1H-pyrrol-3-yl)acetic acid (14b). Physicochemical, spectroscopic, and analytical data are consistent with those reported in the literature [5].

2-(5-(3,4-difluorophenyl)-2-methyl-1-(4-sulfamoylphenyl)-1*H*-pyrrol-3-yl)acetic acid (**14c**). White powder, 75% yield. ^{1}H NMR (400 MHz, DMSO-d_6): δ ppm= 12.20 (s broad, 1H), 7.87 (d, 2H, *J*= 8.5 Hz), 7.49 (s broad, 2H), 7.41 (d, 2H, *J*= 8.5 Hz), 7.30-7.23 (m, 1H), 7.04-6.99 (m, 1H), 6.80-6.77 (m, 1H), 6.39 (s, 1H), 3.40 (s, 2H), 2.01 (s, 3H).

2-(2-methyl-1-(4-sulfamoylphenyl)-5-(*p*-tolyl)-1*H*-pyrrol-3-yl)acetic acid (**14d**). White powder, 70% yield. ^{1}H NMR (400 MHz, DMSO-d_6): δ ppm= 12.12 (s broad, 1H), 7.84 (d, 2H, *J*= 8.5 Hz), 7.47 (s broad, 2H), 7.37 (d, 2H, *J*= 8.5 Hz), 6.99 (d, *J* = 8.1 Hz, 2H), 6.89 (d, *J* = 8.1 Hz, 2H), 6.25 (s, 1H), 3.39 (s, 2H), 2.21 (s, 3H), 2.01 (s, 3H).

2-(1-(3-fluorophenyl)-2-methyl-5-(4-sulfamoylphenyl)-1H-pyrrol-3-yl)acetic acid (14e). Physicochemical, spectroscopic, and analytical data are consistent with those reported in the literature [1].

General procedure for the preparation of propargylic esters 15a-b, 18 and 20.

To a solution of the appropriate acid (**14a-b**, **17** and **19**) (0.77 mmol) in *N*,*N*-dimethylformamide (DMF) (3 mL), potassium carbonate (0.18 g) and propargyl bromide 80% solution in toluene (0.2

mL) were added at room temperature under a nitrogen atmosphere. The mixture was then heated at 40 °C and stirred for 1 h. After removal of the solvent under vacuum, the mixture was diluted with water and extracted with ethyl acetate. The organic phases were then washed with 1N HCl, brine and dried over Na_2SO_4. After filtration and concentration under reduced pressure, the crude product was purified on silica gel using a mixture of cyclohexane/ethyl acetate (3:1 (v/v)) as eluent to afford derivatives **15a-b, 18** and **20** in good yields (37-90%).

Prop-2-yn-1-yl 2-(1-(3-fluorophenyl)-2-methyl-5-(4-(methylsulfonyl)phenyl)-1*H*-pyrrol-3-yl)acetate (**15a**). Yellow powder, 80% yield. ^{1}H NMR ($CDCl_3$): δ ppm= 7.68 (d, 2H, *J*= 8.6 Hz), 7.41-7.35 (m, 1H), 7.17 (d, 2H, *J*= 8.6 Hz), 7.13-7.08 (m, 1H), 6.96-6.94 (m, 1H), 6.92-6.89 (m, 1H), 6.52 (s, 1H), 4.74 (d, 2H, *J*= 2.5 Hz), 3.57 (s, 2H), 3.01 (s, 3H), 2.50 (t, 1H, *J*= 2.5 Hz), 2.09 (s, 3H).

Prop-2-yn-1-yl 2-(1-(4-fluorophenyl)-2-methyl-5-(4-(methylsulfonyl)phenyl)-1*H*-pyrrol-3-yl)acetate (**15b**). Yellow powder, 92% yield. ^{1}H NMR ($CDCl_3$): δ ppm= 7.67 (d, 2H, *J*= 8.5 Hz), 7.17-7.08 (m, 6H), 6.52 (s, 1H), 4.74 (d, 2H, *J*= 2.5 Hz), 3.57 (s, 2H), 3.02 (s, 3H), 2.50 (t, 1H, *J*= 2.5 Hz), 2.07 (s, 3H).

Prop-2-yn-1-yl 1-(4-fluorophenyl)-5-(4-(methylsulfonyl)phenyl)-1*H*-pyrrole-2-carboxylate (**18**). Yellow powder, 37% yield. ^{1}H NMR (400 MHz, $CDCl_3$): δ ppm= 7.76 (d, 2H, *J*= 8.5 Hz), 7.28-7.25 (m, 3H), 7.20-7.17 (m, 2H), 7.09-7.05 (m, 2H), 6.55 (d, 1 H, *J* = 4.1 Hz), 4.73 (d, 2H, *J* = 2.5 Hz), 3.02 (s, 3H), 2.46 (t, 1H, *J* = 2.5 Hz).

Prop-2-yn-1-yl 4-(2-methyl-5-(*p*-tolyl)-1*H*-pyrrol-1-yl)benzoate (**20**). Yellow powder, 90% yield. ^{1}H NMR ($CDCl_3$): δ ppm= 8.06 (d, 2H, *J*= 8.3 Hz), 7.22 (d, 2H, *J*= 8.3 Hz), 6.95 (d, 2H, *J*= 8.1 Hz), 6.91 (d, 2H, *J*= 8.1 Hz), 6.32 (d, 1H, *J*= 3.3 Hz), 6.11 (d, 1H, *J*= 3.3 Hz), 4.93 (d, 2H, *J*= 2.2 Hz), 2.53 (t, 1H, *J*= 2.5 Hz), 2.26 (s, 3H), 2.16 (s, 3H).

General procedure for the preparation of propargylic esters 15c-e.

To a solution of the appropriate acid (**14c-e**) (1 mmol) in *N*,*N*-dimethylformamide (DMF) (3 mL), TEA (1.2 mmol), DMAP (1.2 mmol) and EDCI (1.2 mmol) were added in sequence under a nitrogen atmosphere. The mixture was stirred for 1 h at room temperature, then propargyl alcohol (4 mmol) was added. After 15 h the reaction was quenched with water (10 mL) and extracted with DCM. The organic layers were then washed with 1N HCl, brine and dried over Na_2SO_4. After filtration and evaporation under vacuum, the crude product was purified on silica gel using a mixture of cyclohexane/ethyl acetate (1:1 (v/v)) as eluent to afford derivatives **15c-e** as yellowish solids.

Prop-2-yn-1-yl 2-(5-(3,4-difluorophenyl)-2-methyl-1-(4-sulfamoylphenyl)-1*H*-pyrrol-3-yl)acetate (**15c**). Yellow powder, 25% yield. ^{1}H NMR (400 MHz, $CDCl_3$): δ ppm= 7.95 (d, 2H, *J*= 8.5 Hz), 7.26 (d, 2H, *J*= 8.5 Hz), 6.97-6.90 (m, 1H), 6.81-6.78 (m, 1H), 6.69-6.67 (m, 1H), 6.35 (s, 1H), 5.05 (s broad, 2H), 4.73 (d, 2H, *J* = 2.3 Hz), 3.55 (s, 2H), 2.49 (t, 1H, *J* = 2.3 Hz), 2.08 (s, 3H).

Prop-2-yn-1-yl 2-(2-methyl-1-(4-sulfamoylphenyl)-5-(*p*-tolyl)-1*H*-pyrrol-3-yl)acetate (**15d**): yellow powder, 25% yield. ^{1}H NMR (400 MHz, $CDCl_3$) δ ppm: 7.89 (d, 2H, *J*= 8.6 Hz), 7.25 (d, 2H, *J*= 8.6 Hz), 6.95 (d, 2H, *J* = 8.1 Hz), 6.88 (d, 2H, *J* = 8.1 Hz), 6.33 (s, 1H), 5.00 (s broad, 2H), 4.73 (d, 2H, *J* = 2.5 Hz), 3.56 (s, 2H), 2.48 (t, 1H, *J* = 2.5 Hz), 2.25 (s, 3H), 2.09 (s, 3H).

Prop-2-yn-1-yl 2-(1-(3-fluorophenyl)-2-methyl-5-(4-sulfamoylphenyl)-1*H*-pyrrol-3-yl)acetate (15e): yellow powder, 26% yield. ^{1}H NMR (400 MHz, $CDCl_3$) δ ppm: 7.67 (d, 2H, *J*= 8.5 Hz), 7.40-7.35 (m, 1H), 7.14-7.08 (m, 3H), 6.96-6.88 (m, 2H), 6.50 (s, 1H), 4.80 (s, 2H), 4.75-4.73 (m, 4H), 3.57 (s, 2H), 2.50 (t, 1H, *J* = 2.5 Hz), 2.09 (s, 3H).

General procedure for the synthesis of 1-(3-fluorophenyl)-2-methyl-5-(4-((3-(trimethylsilyl)propyl)sulfonyl)phenyl)-1H-pyrrole (16).

Compound **16** was prepared according to the same procedure outlined previously [Consalvi2015]. Lithium diisopropylamide (LDA) was prepared *in situ* by adding dropwise 3.9 mL of butyllithium solution (2.5 M in hexane) to a solution of diisopropylamine (DIPA) (1.75 mL) in dry THF (13 mL) at 0 °C and under nitrogen atmosphere. After 30 minutes stirring, the reaction was cooled down to -78 °C and a solution of the pyrrole **11a** (8 mmol) in dry THF (23 mL) was slowly added. The mixture was stirred for 1.5 h. 2.58 mL of iodomethyltrimethylsilane were then added dropwise and the reaction was allowed to warm to room temperature. After 15h, the reaction was quenched with water and the pH was adjusted (pH= 2) with 1N HCl. The so obtained mixture was extracted with ethyl acetate and the organic layers were washed with brine and dried over Na_2SO_4. After evaporation under vacuum, the crude material was purified on silica gel using a mixture of cyclohexane/ethyl acetate 15:1 (v/v) to afford **16** as a white powder.

1-(3-fluorophenyl)-2-methyl-5-(4-((3-(trimethylsilyl)propyl)sulfonyl)phenyl)-1H-pyrrole (16). Physicochemical, spectroscopic, and analytical data are consistent with those reported in the literature [1].

General procedure for the preparation of 4-(1-(3-fluorophenyl)-5-methyl-1*H*-pyrrol-2-yl)benzenesulfonamide (11e)

A solution of tetrabutylammonium fluoride (5 mL, 1M in THF) was added to a solution of compound **16** (1.5 mmol) in dry THF (12 mL) under a nitrogen flow and refluxed for 1.5 h. After cooling down to room temperature, a solution of sodium acetate (1.3 g) and hydroxylamine-*O*-sulfonic acid (1.8 g) in water (5 mL) was added, and the reaction was stirred for 1 h. The mixture was then diluted with water and extracted with ethyl acetate. The organic layers were washed with $NaHCO_3$ saturated solution, brine and dried over Na_2SO_4. After filtration and removal of the solvent under vacuum, the crude product was purified on silica gel with a mixture of cyclohexane/ethyl acetate 2:1 (v/v) as eluent. Recrystallization from diethyl ether yielded **11e** as a white powder.

4-(1-(3-fluorophenyl)-5-methyl-1*H*-pyrrol-2-yl)benzenesulfonamide (11e). Physicochemical, spectroscopic, and analytical data are consistent with those reported in the literature [1].

General procedure for the preparation of lithium salts 23a-b.

A solution of lithium bis(trimethylsilyl)amide (12.3 mmol) in 30 mL of anhydrous THF was cooled to -78°C. Afterwards, a solution of the appropriate acetophenone **21a-b** in 3 ml of anhydrous THF was added dropwise and the mixture was stirred for 1h. Diethyl oxalate was then added over 5 minutes and the resulting dark orange solution was warmed to room temperature over 4 h. After 18 h stirring, the precipitate was filtered off and washed with diethyl ether affording lithium salts **23a-b** in 80% yield.

Lithium (*Z*)-1-ethoxy-4-(4-isopropylphenyl)-1,4-dioxobut-2-en-2-olate (23a). Physicochemical, spectroscopic, and analytical data are consistent with those reported in the literature [2].

Lithium (*Z*)-1-ethoxy-1,4-dioxo-4-(4-(trifluoromethyl)phenyl)but-2-en-2-olate (23b). Physicochemical, spectroscopic, and analytical data are consistent with those reported in the literature [2].

General procedure for the preparation of carboxylates 24a-b

4-fluorophenylhydrazine hydrochloride (2.12 mmol) was added to a solution of the suitable lithium salt **23a-b** (2.12 mmol) in 12 mL of ethanol. The reaction mixture was heated at 90°C for 5 h and then cooled down to room temperature. After removal of the solvent under vacuum, the mixture was extracted with ethyl acetate and the organic layers were washed with brine, dried over Na_2SO_4 and evaporated *in vacuo*. The crude product was purified by column chromatography (cyclohexane/ethyl acetate 2/1 (v/v)) to yield carboxylates **24a-b** in good yields (65-70%).

Ethyl 1-(4-fluorophenyl)-5-(4-isopropylphenyl)-1*H*-pyrazole-3-carboxylate (24a). Physicochemical, spectroscopic, and analytical data are consistent with those reported in the literature [2].

Ethyl 1-(4-fluorophenyl)-5-(4-(trifluoromethyl)phenyl)-1*H*-pyrazole-3-carboxylate (24b). Physicochemical, spectroscopic, and analytical data are consistent with those reported in the literature [2].

General procedure for the preparation of alcohols 25a-b

The appropriate carboxylate **24a-b** (0.41 mmol) was dissolved in THF and cooled down to 0°C, then $LiAlH_4$ was added dropwise under a nitrogen flow (0.49 mL, 1 M in THF). At the end, the reaction was cooled down to 0°C and quenched with 0.18 mL of ethyl acetate, 0.08 mL of water and 0.1 mL of NaOH 2N. After 30 minutes stirring, the precipitate was filtered off and the filtrate was dried over Na_2SO_4 and concentrated *in vacuo*. The resulting crude product was purified by column chromatography (cyclohexane/ethyl acetate 1/1 (v/v)) to afford alcohols **25a-b** in good yields (63-91%).

(1-(4-Fluorophenyl)-5-(4-isopropylphenyl)-1*H*-pyrazol-3-yl)methanol (25a). Physicochemical, spectroscopic, and analytical data are consistent with those reported in the literature [2].

(1-(4-Fluorophenyl)-5-(4-(trifluoromethyl)phenyl)-1*H*-pyrazol-3-yl)methanol (25b). Physicochemical, spectroscopic, and analytical data are consistent with those reported in the literature [2].

General procedure for the preparation of propargylic ethers 26a-b

A solution of the appropriate alcohol **25a-b** (0.22 mmol) in THF (2 mL) was added dropwise at 0°C to a suspension of NaH 60% (0.22 mmol) in mineral oil under a nitrogen atmosphere. After stirring for 10 min, the mixture was allowed to warm at room temperature, and an 80% solution of propargyl bromide in toluene (0.66 mmol) was added. After 24 h, the reaction was cooled down to 0 °C, quenched with water (10 mL) and stirred for 30 minutes. The resulting mixture was extracted with ethyl acetate and the organic layers were washed with a $NaHCO_3$ saturated solution, brine and dried over Na_2SO_4. After filtration and removal of the solvent under vacuum, the solid residue was purified on silica gel using a mixture of petroleum ether/ethyl acetate (10:1 (v/v)) as eluent to afford the desired products **26a-b** in good yields (65-70%).

1-(4-fluorophenyl)-5-(4-isopropylphenyl)-3-((prop-2-yn-1-yloxy)methyl)-1*H*-pyrazole (**26a**). Colorless oil, 65% yield. ^{1}H NMR ($CDCl_3$): δ ppm= 7.29-7.26 (m, 2H), 7.17-7.11 (m, 4H), 7.04-7.00 (m, 2H), 6.53 (s, 1H), 4.70 (s, 2H), 4.29 (d, 2H, *J*= 2.4 Hz), 2.89 (sept, 1H, *J*= 6.9 Hz), 2.48 (t, 1H, *J*= 2.4 Hz), 1.24 (d, 6H, *J*= 6.9 Hz).

1-(4-fluorophenyl)-3-((prop-2-yn-1-yloxy)methyl)-5-(4-(trifluoromethyl)phenyl)-1*H*-pyrazole (**26b**). Colorless oil, 70% yield. ^{1}H NMR ($CDCl_3$) δ ppm: 7.57 (d, 2H, *J*= 8.3 Hz), 7.33 (d, 2H, *J*= 8.3 Hz), 7.27-7.04 (m, 2H), 7.08-7.04 (m, 2H), 6.63 (s, 1H), 4.72 (s, 2H), 4.30 (d, 2H, *J*= 2.3 Hz), 2.49 (t, 1H, *J*= 2.3 Hz).

General procedure for the synthesis of (dicobalthexacarbonyl)derivatives 1-9

To a solution of the appropriate propargyl derivative (**15a-e**, **18**, **20** and **26a-b**) (0.62 mmol) dissolved in the least volume of THF, dicobalt octacarbonyl (0.68 mmol) was added under a nitrogen atmosphere at room temperature. The resulting black mixture was left stirring for 2 h and the solvent was removed under reduced pressure. The so obtained black crude product was filtered on celite and then thoroughly washed with DCM. The filtrate was concentrated *in vacuo* and the black residue was purified on silica gel with a mixture of cyclohexane/ethyl acetate (20:1 (v/v)) or a mixture of DCM/ethyl acetate (15:1 (v/v)) as the mobile phase to obtain final products **1-9** as red solids.

Prop-2-yn-1-yl 2-(1-(4-fluorophenyl)-2-methyl-5-(4-(methylsulfonyl)phenyl)-1*H*-pyrrol-3-yl)acetate hexacarbonyldicobalt (**1**). Red powder, 80% yield. ^{1}H NMR (400 MHz, $CDCl_3$): δ ppm= 7.60 (d, *J* = 8.1 Hz, 2H), 7.16-7.12 (m, 6H), 6.52 (s, 1H), 6.08 (s, 1H), 5.34 (s, 2H), 3.60 (s, 2H), 3.01 (s, 3H), 2.06 (s, 3H). ^{13}C NMR (100 MHz, $CDCl_3$): δ ppm= 202.3, 171.7, 164.28, 161.53,138.30,

136.88, 134.88, 134.32, 130.07, 129.59, 121.07, 120.47, 116.56, 113.37, 110.08, 87.99, 71.76, 65.24, 44.48, 31.91, 10.92.

Prop-2-yn-1-yl 2-(1-(3-fluorophenyl)-2-methyl-5-(4-(methylsulfonyl)phenyl)-1*H*-pyrrol-3-yl)acetate hexacarbonyldicobalt (**2**). Red powder, 80% yield. ^{1}H NMR (400 MHz, $CDCl_3$): δ ppm= 7.68 (d, 2H, *J* = 8.1 Hz), 7.41-7.35 (m, 1H), 7.17 (d, 2H, *J* = 8.1 Hz), 7.14-7.10 (m, 1H), 6.96-6.88 (m, 2H), 6.53 (s, 1H), 6.08 (s, 1H), 5.34 (s, 2H), 3.60 (s, 2H), 3.01 (s, 3H), 2.09 (s, 3H). ^{13}C NMR (100 MHz, $CDCl_3$): δ ppm= 199.21, 171.60, 164.04, 161.53, 140.40, 140.37, 140.29, 138.14, 137.01, 131.62, 131.37, 130.67, 130.61, 127.38, 124.40, 116.07, 115.55, 115.23, 113.75, 112.79, 88.80, 72.05, 65.27, 44.20, 31.88, 10.95.

Prop-2-yn-1-yl 2-(5-(3,4-difluorophenyl)-2-methyl-1-(4-sulfamoylphenyl)-1H-pyrrol-3-yl)acetate hexacarbonyldicobalt (**3**). Red powder, 10% yield. ^{1}H NMR (400 MHz, DMSO-d_6): δ ppm= 7.87 (d, *J* = 8.1 Hz, 2H), 7.50 (s broad, 2H), 7.40 (d, *J* = 8.1 Hz, 2H), 7.30-7.23 (m, 1H), 7.02-6.97 (m, 1H), 6.80-6.78 (m, 2H), 6.38 (s, 1H), 5.39 (s, 2H), 3.57 (s, 2H), 2.01 (s, 3H). ^{13}C NMR (100 MHz, $CDCl_3$) δ ppm: 201.57, 171.22, 152.88, 151.24, 147.97, 142.74, 141.08, 131.35, 129.01, 127.60, 123.98, 123.88, 117.26, 117.09, 116.79, 116.61, 113.53, 111.84, 89.52, 75.06, 65.42, 32.01, 11.16.

Prop-2-yn-1-yl 2-(2-methyl-1-(4-sulfamoylphenyl)-5-(*p*-tolyl)-1*H*-pyrrol-3-yl)acetate hexacarbonyldicobalt (**4**). Red powder, 40% yield. ^{1}H NMR (400 MHz, DMSO-d_6): δ ppm= 7.84 (d, 2H, *J* = 8.2 Hz), 7.49 (s broad, 2H), 7.36 (d, 2 H, *J* = 8.2 Hz), 7.00 (d, 2H, *J* = 8 Hz), 6.88 (d, 2H, *J* = 8 Hz), 6.79 (s, 1H), 6.25 (s, 1H), 5.39 (s, 2H), 3.56 (s, 2H), 2.21 (s, 3H), 2.01 (s, 3H). ^{13}C NMR (100 MHz, $CDCl_3$); δ ppm= 201.37, 171.38, 143.35, 140.46, 136.08, 133.59, 129.63, 129.07, 128.99, 128.54, 127.96, 127.33, 113.26, 110.80, 89.02, 75.00, 62.29, 32.16, 21.06, 11.22.

Prop-2-yn-1-yl 2-(1-(3-fluorophenyl)-2-methyl-5-(4-sulfamoylphenyl)-1*H*-pyrrol-3-yl)acetate hexacarbonyldicobalt (**5**). Red powder, 40% yield. ^{1}H NMR (400 MHz, DMSO-d_6): δ ppm= 7.60 (d, 2H, *J* = 8.1 Hz), 7.53–7.47 (m, 1H), 7.32–7.20 (m, 4H), 7.14 (d, 2H, *J* = 8.1 Hz), 7.04-7.02 (m, 1H), 6.79 (s, 1H), 6.47 (s, 1H), 5.39 (s, 2H), 3.58 (s, 2H), 2.03 (s, 3H). ^{13}C NMR (100 MHz, $CDCl_3$): δ ppm= 200.05, 171.26, 163.07, 160.56, 138.47, 137.16, 131.46, 131.32, 130.66, 127.35, 126.41, 124.41, 116.02, 115.80, 115.22, 113.42, 112.59, 88.63, 75.09, 65.37, 32.03, 11.08.

Prop-2-yn-1-yl 1-(4-fluorophenyl)-5-(4-(methylsulfonyl)phenyl)-1*H*-pyrrole-2-carboxylate hexacarbonyldicobalt (6). Red powder, 90% yield. ^{1}H NMR (400 MHz, $CDCl_3$): δ ppm= 7.76 (d, 2H, *J*= 8.1 Hz), 7.28-7.25 (m, 3H), 7.20-7.17 (m, 2H), 7.10-7.06 (m, 2H), 6.55 (d, 1 H, *J* = 3.7 Hz), 6.08 (s, 1H), 5.31 (s, 2H), 3.02 (s, 3H). ^{13}C NMR (100 MHz, $CDCl_3$) δ ppm: 201.7, 170.4, 163.02, 160.13, 140.31, 138.99, 135.23, 135.01, 130.12, 128.59, 128.33, 127.01, 122.98, 121.14, 117.01, 112.35, 88.43, 76.17, 56.24, 43.98.

Prop-2-yn-1-yl 4-(2-methyl-5-(*p*-tolyl)-1*H*-pyrrol-1-yl)benzoate hexacarbonyldicobalt (**7**). Red powder, 70% yield. ^{1}H NMR (400 MHz, $CDCl_3$): δ ppm= 8.09 (d, 2H, *J*= 8.5 Hz), 7.21 (d, 2H, *J*= 8.5 Hz), 6.95-6.89 (m, 4H), 6.32 (d, 1H, *J*= 3.2 Hz), 6.13 (s, 1H), 6.10 (d, 1H, *J*= 3.2 Hz), 5.53 (s, 2H), 2.26 (s, 3H), 2.16 (s, 3H). ^{13}C NMR (100 MHz, $CDCl_3$): δ ppm= 199.97, 170.13, 141.52, 139.11, 134.00, 130.11, 127.73, 127.64, 127.02, 126.83, 126.34, 122.16, 112.83, 111.96, 89.75, 78.06, 56.28, 21.47, 12.01.

1-(4-fluorophenyl)-5-(4-isopropylphenyl)-3-((prop-2-yn-1-yloxy)methyl)-1*H*-pyrazole hexacarbonyldicobalt (8). Red powder, 70% yield. ^{1}H NMR (400 MHz, $CDCl_3$): δ ppm= 7.27–7.23 (m, 2H), 7.17–7.09 (m, 4H), 7.04-7.00 (m, 2H), 6.54 (s, 1H), 6.09 (s, 1H), 4.78 (m, 4H), 2.90 (sept, 1H, *J* = 7.0 Hz), 1.24 (d, 6H, *J* = 7.0 Hz). ^{13}C NMR (100 MHz, $CDCl_3$): δ ppm= 200.12, 162.12, 159.04, 149.25, 145.03, 136.27, 130.11, 128.82, 127.54, 127.35, 126.01, 115.92, 115.69, 106.76, 89.16, 76.80, 65.13, 58.09, 33.81, 23.45.

1-(4-fluorophenyl)-3-((prop-2-yn-1-yloxy)methyl)-5-(4-(trifluoromethyl)phenyl)-1H-pyrazole hexacarbonyldicobalt (9): Red powder, 63% yield. ^{1}H NMR (400 MHz, $CDCl_3$): δ ppm= 7.57 (d, 2H, *J* = 8.2 Hz), 7.29 (d, 2H, *J* = 8.2 Hz), 7.25–7.22 (m, 2H), 7.08-7.04 (m, 2H), 6.63 (s, 1H), 6.10 (s, 1H), 4.80 (m, 4H). ^{13}C NMR (100 MHz, $CDCl_3$): δ ppm= 200.03, 163.01, 160.46, 147.98, 142.03, 136.30, 135.82, 130.78, 130.51, 130.21, 130.01, 128.78, 127.26, 123.58, 123.19, 121.64, 116.48, 116.20, 108.13, 89.88, 77.20, 65.50, 59.03.

References

1. Consalvi, S.; Alfonso, S.; Di Capua, A.; Poce, G.; Pirolli, A.; Sabatino, M.; Ragno, R.; Anzini, M.; Sartini, S.; La Motta, C.; et al. Synthesis, Biological Evaluation and Docking Analysis of a New Series of Methylsulfonyl and Sulfamoyl Acetamides and Ethyl Acetates as Potent COX-2 Inhibitors. *Bioorg Med Chem* **2015**, *23*, 810–820, doi:10.1016/j.bmc.2014.12.041.
2. Poce, G.; Consalvi, S.; Venditti, G.; Alfonso, S.; Desideri, N.; Fernandez-Menendez, R.; Bates, R.H.; Ballell, L.; Barros Aguirre, D.; Rullas, J.; et al. Novel Pyrazole-Containing Compounds Active against Mycobacterium Tuberculosis. *ACS Med Chem Lett* **2019**, *10*, 1423–1429, doi:10.1021/acsmedchemlett.9b00204.
3. Biava, M.; Porretta, G.C.; Poce, G.; Supino, S.; Deidda, D.; Pompei, R.; Molicotti, P.; Manetti, F.; Botta, M. Antimycobacterial Agents. Novel Diarylpyrrole Derivatives of BM212 Endowed with High Activity toward Mycobacterium Tuberculosis and Low Cytotoxicity. *J Med Chem* **2006**, *49*, 4946–4952, doi:10.1021/jm0602662.
4. Biava, M.; Porretta, G.C.; Poce, G.; Supino, S.; Forli, S.; Rovini, M.; Cappelli, A.; Manetti, F.; Botta, M.; Sautebin, L.; et al. Cyclooxygenase-2 Inhibitors. 1,5-Diarylpyrrol-3-Acetic Esters with Enhanced Inhibitory Activity toward Cyclooxygenase-2 and Improved Cyclooxygenase-2/Cyclooxygenase-1 Selectivity. *J Med Chem* **2007**, *50*, 5403–5411, doi:10.1021/jm0707525.
5. Biava, M.; Porretta, G.C.; Poce, G.; Battilocchio, C.; Manetti, F.; Botta, M.; Forli, S.; Sautebin, L.; Rossi, A.; Pergola, C.; et al. Novel Ester and Acid Derivatives of the 1,5-Diarylpyrrole Scaffold as Anti-Inflammatory and Analgesic Agents. Synthesis and in Vitro and in Vivo Biological Evaluation. *J Med Chem* **2010**, *53*, 723–733, doi:10.1021/jm901269y.
